# A new neodymium complex on renewable magnetic biochar nanoparticles as an environmentally friendly, recyclable and efficient nanocatalyst in the homoselective synthesis of tetrazoles†

**DOI:** 10.1039/d3na01087b

**Published:** 2024-02-26

**Authors:** Bahman Tahmasbi, Parisa Moradi, Mitra Darabi

**Affiliations:** a Department of Chemistry, Faculty of Science, Ilam University PO Box 69315516 Ilam Iran b.tahmasbi@ilam.ac.ir bah.tahmasbi@gmail.com

## Abstract

Inexpensive, stable, selective, and recyclable nanocatalysts, waste regeneration, and utilization of safe and available solvents are of interest and important factors in laboratory science and industrial applications of green chemistry. Therefore, herein, biochar nanoparticles (BNPs) were synthesized through chicken manure pyrolysis as a novel method for waste recycling. Then, in order to improve their recyclability, the obtained BNPs were magnetized using magnetic nickel nanoparticles. Then, the surface of the biochar magnetic nanoparticles (BMNPs) was modified by (3-chloropropyl)trimethoxysilane (3-CPTMS) and further a novel neodymium Schiff-base complex was immobilized on the surface of the modified BMNPs, denoted as Nd-Schiff-base@BMNPs. The obtained supported neodymium complex was used as a practical, selective, biocompatible, commercial, and reusable heterogeneous nanocatalyst. The biochar support of this nanocatalyst was formed from pyrolysis of chicken manure; therefore, it is cheap, economically viable, green and also compatible with the principles of green chemistry. Nd-Schiff-base@BMNPs acts selectively in organic reactions and also it can easily be recovered using an external magnet and reused, which is compatible with the principles of green chemistry. This nanocatalyst was characterized by wavelength-dispersive X-ray spectroscopy (WDX), scanning electron microscopy (SEM), energy-dispersive X-ray spectroscopy (EDS), thermogravimetric analysis (TGA), Fourier transform infrared spectroscopy (FT-IR), inductively coupled plasma (ICP), and N_2_ adsorption–desorption (BET method) techniques. In the next step, the catalytic utilization of Nd-Schiff-base@BMNPs was investigated in the homoselective synthesis of 5-substituted 1*H*-tetrazole compounds from [3 + 2] cycloaddition of sodium azide (NaN_3_) and organo-nitriles in PEG-400 as a green solvent. Utilizing PEG-400 as a solvent offers various advantages, *e.g.* cheap, readily available, and environmentally friendly solvent as well as rapid separation and high purity of products. Therefore, this work is fully compatible with the principles of green chemistry.

## Abstract 2

A new heterogeneous catalyst of neodymium Schiff-base complex was immobilized on biochar magnetic nanoparticles, and then, it was used as a practical and reusable nanocatalyst for the homoselective synthesis of tetrazoles.

## Graphical abstract

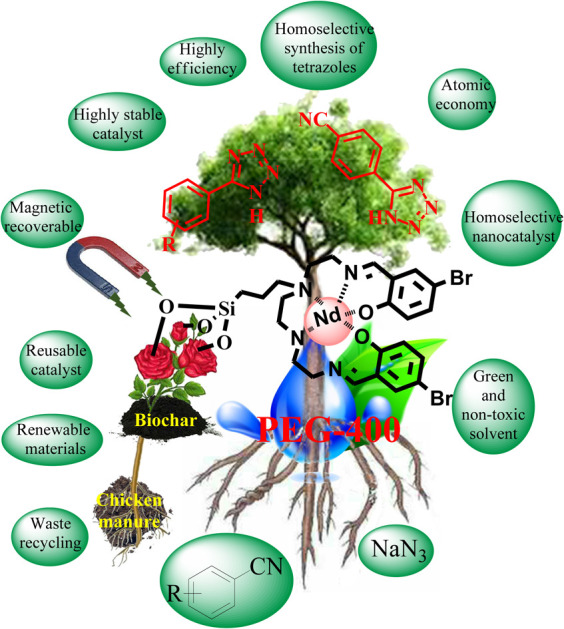

## 1. Introduction

During the last few decades, the use of Earth's mineral and non-renewable resources has greatly increased in industrial and laboratory applications, which is a serious risk for the future. For example, oxides of precious metals such as iron, aluminum, titanium, zinc, cobalt, tungsten, *etc.* and other inorganic compounds have been reported as catalyst supports in numerous articles.^1–6^ Meanwhile, modern chemistry and green chemistry emphasize the use of renewable materials, so that one of the principles of green chemistry is waste recycling.^7–13^ Therefore, in order to recycle agricultural waste, in this work we synthesized biochar nanoparticles from pyrolysis of chicken manure and used them as a recyclable support for the synthesis of a reusable and heterogeneous catalyst. This is because another principle of green chemistry is the use of reusable, cheap and sustainable catalysts.^14^ Homogeneous and heterogeneous catalysts are two main categories of catalysts that have advantages and disadvantages. Meanwhile, the difficult recycling of homogeneous catalysts and the low catalytic activity of heterogeneous catalysts are two main challenges.^15–18^ Therefore, nanocatalysts as a bridge between homogeneous and heterogeneous catalysts have recently received attention because they have high catalytic activity and are easily recycled and reused, so they do not have the disadvantages of conventional catalysts, while having their own advantages.^19–22^ In this context, different nanoparticles such as mesoporous silica materials,^23–25^ graphene oxide,^26^ polymers,^27,28^ hercynite,^29^ MOF nanoparticles,^30–32^ boehmite,^33–36^ magnetic nanoparticles,^37,38^ carbon nanostructures,^39,40^ biochar,^41–45^*etc.* have been reported as catalysts or catalyst supports. Except biochar nanoparticles, all these nanoparticles are produced from non-renewable sources. In fact, biochar is carbon black that is synthesized from pyrolysis of agricultural waste at high temperature.^41^ Therefore, biochar nanoparticles are stable at high temperature, in air atmosphere, and in various solutions.^46^ Also, biochar nanoparticles are inexpensive and environmentally friendly. In addition, the high density of COOH, C=O and OH groups on the surface of biochar can establish a high surface activity for biochar nanoparticles,^46–52^ which provide for modification of their surface and high loading of immobilized catalysts. Therefore, biochar nanoparticles can be a suitable alternative for catalyst supports that are made from non-renewable materials.

Despite several studies on biochar application and its properties,^53–55^ biochar nanoparticles have rarely been reported as catalysts or catalyst supports.^50^ However, recovering and reusing biochar nanoparticles need difficult and time-consuming methods such as filtration and centrifugation. Magnetization of biochar can address these challenges, because such magnetic nanoparticles are easily separated by applying an external magnet. Therefore, we report a Schiff-base complex of neodymium on biochar magnetic nanoparticles (Nd-Schiff-base@BMNPs) as a highly practical, biocompatible and recyclable nanocatalyst for the homoselective synthesis of 5-substituted 1*H*-tetrazole compounds from [3 + 2] cycloaddition of NaN_3_ and organo-nitriles in PEG-400 as solvent. PEG is a green and non-toxic solvent, because, based on another principle of green chemistry, the use of auxiliary substances such as solvents should be made innocuous.^56,57^ Another principle of green chemistry is atomic economy.^58^ Synthesis of 5-substituted 1*H*-tetrazole compounds from [3 + 2] cycloaddition of NaN_3_ and organo-nitriles is a completely atom-economic process, because all the atoms of the starting materials are present in the products. Also, tetrazoles are a large class of organic materials which have many usages in different fields such as medicinal chemistry, drugs, synthetic organic chemistry, coordination chemistry, catalysis technology, photographic industry, green explosives and as effective stabilizers of metallopeptide structures.^58–61^ For example, candesartan, TAK-456, valsartan, irbesartan, losartan, olmesartan medoxomil, cilostazol, pemirolast and pranlukast are various pharmacological applications of tetrazole derivatives.^59–61^

## 2. Experimental

### 2.1. Synthesis of the Schiff base ligand

**Scheme 1:**
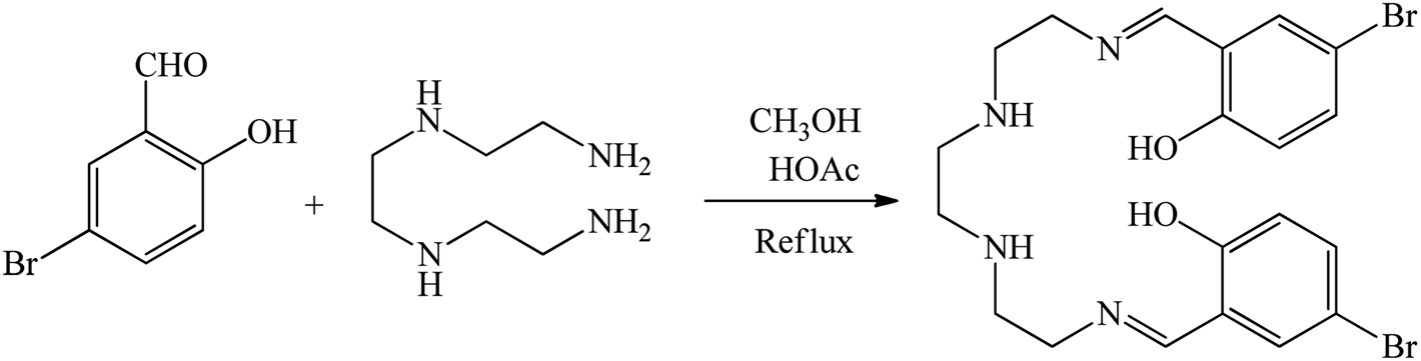
Synthesis of 2,2′-((1*E*,11*E*)-2,5,8,11-tetraazadodeca-1,11-diene-1,12-diyl)bis(4-bromophenol) as Schiff base ligand.

Initially, 2,2′-((1*E*,11*E*)-2,5,8,11-tetraazadodeca-1,11-diene-1,12-diyl)bis(4-bromophenol) was produced from condensation of triethylenetetramine (TETA) (1 mL) and 5-bromosalicylaldehyde (5-BrSA) (2.693 g) in MeOH, which is defined as the Schiff base ligand in this work (Scheme 1). A solution of TETA was injected drop by drop into a solution of 5-BrSA under stirring in the presence of acetic acid (4 drops). The reaction mixture was refluxed for 4 h. The synthesized yellow precipitate was filtered, washed with MeOH, and then dried at r.t.^62^ As is known, bromine acts as an electron-donating group by sharing non-bonding electron pairs through resonance. While due to the high electronegativity of bromine, it acts as an electron-withdrawing group through induction effects. In 5-BrSA, bromine is in the *meta* position with respect to carbaldehyde, which acts as an electron-withdrawing group for carbaldehyde. Therefore, it facilitates the formation of the Schiff-base ligand. In addition, in 5-BrSA, bromine is in the *para* position with respect to the hydroxyl group, which acts as an electron-donating group for the hydroxyl group. Therefore, bromine facilitates the coordination of the hydroxyl group with the metal and the formation of the complex. For these reasons, we chose 5-BrSA.

### 2.2. Preparation of biochar magnetic nanoparticles (BMNPs)

BNPs were synthesized by pyrolysis of chicken manure. In this regard, the pyrolysis temperature for 500 g of dried chicken manure was selected as 400 °C. The pyrolysis process was carried out under a nitrogen gas flow rate of 0.03 L min^−1^ for 1 h. After the pyrolysis process, the vessel was cooled with N_2_ sweeping at 0.3 L min^−1^ for 30 min. Then, the obtained BNPs were magnetized by doping with magnetic nickel nanoparticles (MNiNPs).^50^

MNiNPs were prepared by stirring NiCl_2_·6H_2_O (0.5 g) in ethylene glycol (EG) (30 mL) at 60 °C. Then, 1.4 mL of hydrazine hydrate was injected drop by drop. Finally, 3.6 mL of sodium hydroxide (1 M) solution was injected. After 1 h, the obtained black precipitate was isolated by magnetic decantation and washed with H_2_O.^26^

BNPs (0.3 g) were dispersed in 50 mL of H_2_O. Then, MNiNPs (0.1 g) were added and dispersed again. The obtained mixture was stirred at r.t. for 24 h. The obtained BMNPs were isolated by magnetic decantation, washed with H_2_O and dried at 60 °C.^41^

### 2.3. Preparation of Nd-Schiff-base@BMNPs

**Scheme 2:**
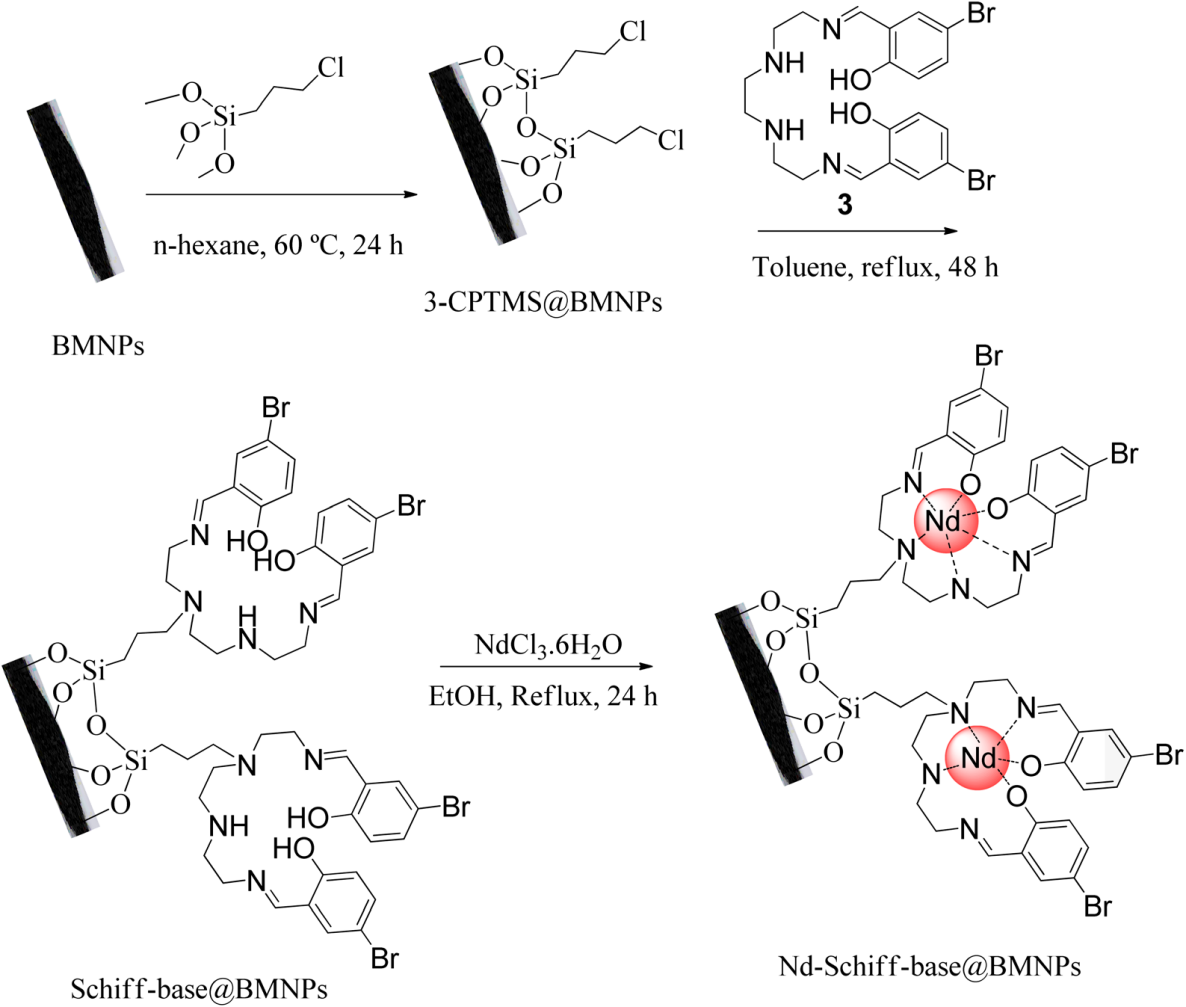
Synthesis of Nd-Schiff-base@BMNPs.

The surface of BMNPs was modified by 3-CPTMS.^50^ As shown in Scheme 2, BMNPs (1 g) were dispersed in 25 mL of *n*-hexane. Then, 1.5 mL of 3-CPTMS was injected into the above mixture and stirred at 60 °C for 24 h under N_2_ atmosphere. The modified BMNPs (3-CPTMS@BMNPs) were isolated by magnetic decantation, washed with EtOH and dried at r.t. Then, 1 g of 3-CPTMS@BMNPs was dispersed in toluene and mixed with 1 mmol of the synthesized Schiff base ligand for 48 h under reflux condition. The magnetic precipitate (Schiff-base@BMNPs) was isolated by magnetic decantation and washed with DMSO and further with EtOH. As the end step, 1 g of Schiff-base@BMNPs was dispersed in EtOH, and mixed with neodymium(iii) chloride hexahydrate (1 mmol) for 24 h under reflux conditions. The obtained catalyst (Nd-Schiff-base@BMNPs) was isolated by magnetic decantation and washed with H_2_O and further with EtOH (Scheme 2).

### 2.4. General system for the synthesis of 5-substituted 1*H*-tetrazoles catalyzed by Nd-Schiff-base@BMNPs

**Scheme 3:**
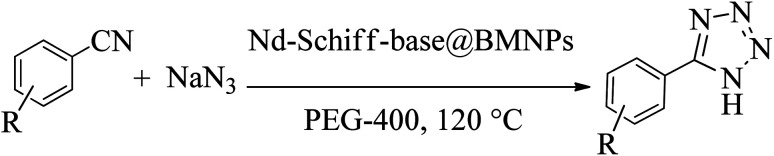
General system for the synthesis of 5-substituted 1*H*-tetrazoles in the presence of Nd-Schiff-base@BMNPs.

In this method, NaN_3_ (0.091 g, 1.4 mmol) and nitrile compounds (1 mmol) were mixed with 50 mg of Nd-Schiff-base@BMNPs and 1 mL of PEG-400 and stirred at 120 °C. After completion of the reaction (monitored by TLC), the mixture was cooled down and was diluted with H_2_O and ethyl acetate (EtOAc). Then, Nd-Schiff-base@BMNPs were isolated *via* magnetic decantation and washed with HCl (4 N, 10 mL). The pH of the solution before adding hydrochloric acid was 6.63, and after adding hydrochloric acid, the pH changed to 1.2. The organic phase (EtOAc), containing the tetrazole product, was isolated by extraction method from H_2_O and it was dried by anhydrous sodium sulphate. Finally, the pure tetrazole products in 89–98% yields were obtained after evaporation of EtOAc (Scheme 3). The obtained tetrazoles were confirmed by ^1^H NMR, ^13^C NMR and FT-IR spectroscopies.

### 2.5. Spectral data

#### 5-Phenyl-1*H*-tetrazole

^1^H NMR (400 MHz, DMSO-d_6_): *δ*_H_ = 8.02 (m, 2H), 7.60 (m, 3H) ppm.

^13^C NMR (100 MHz, DMSO-d_6_): *δ*_C_ = 165.7, 131.6, 129.8, 127.3, 124.5 ppm.

IR (KBr) cm^−1^: 3426, 3128, 3056, 2917, 2864, 2701, 2607, 1852, 1735, 1606, 1559, 1472, 1405, 1248, 1153, 1089, 986, 954, 784, 718, 690, 491.

#### 5-(3-Nitrophenyl)-1*H*-tetrazole

^1^H NMR (400 MHz, DMSO-d_6_): *δ*_H_ = 8.78 (s, 1H), 8.42 (d, *J* = 12 Hz, 1H), 8.37 (d, *J* = 12 Hz, 1H), 7.86 (t, *J* = 12 Hz, 1H) ppm.

^13^C NMR (100 MHz, DMSO-d_6_): *δ*_C_ = 155.3, 148.6, 133.4, 131.5, 126.5, 125.9, 121.8 ppm.

IR (KBr) cm^−1^: 3451, 3306, 3088, 2897, 2753, 1620, 1526, 1347, 1157, 1076, 1013, 913, 866, 820, 735, 452.

#### 2-(1*H*-Tetrazol-5-yl)benzonitrile

^1^H NMR (400 MHz, DMSO-d_6_): *δ*_H_ = 8.07 (t, *J* = 8 Hz, 2H), 7.91 (t, *J* = 8 Hz, 1H), 7.76 (t, *J* = 8 Hz, 1H) ppm.

IR (KBr) cm^−1^: 3397, 2917, 2228, 1732, 1573, 1458, 1357, 1245, 1106, 949, 843, 775, 510.

#### 2-(1*H*-Tetrazol-5-yl)phenol

^1^H NMR (400 MHz, DMSO-d_6_): *δ*_H_ = 7.97 (d, *J* = 12 Hz, 1H), 7.39 (t, *J* = 8 Hz, 1H), 7.05 (d, *J* = 12 Hz, 1H), 7.98 (t, *J* = 8 Hz, 1H), 3.37 (br, 1H) ppm.

^13^C NMR (100 MHz, DMSO-d_6_): *δ*_C_ = 155.7, 152.0, 133.0, 129.4, 120.1, 116.7, 110.9 ppm.

IR (KBr) cm^−1^: 3446, 3254, 3060, 2948, 1712, 1610, 1546, 1476, 1358, 1295, 1230, 1115, 1067, 808, 742, 680, 538, 462.

#### 5-(4-Nitrophenyl)-1*H*-tetrazole

^1^H NMR (400 MHz, DMSO-d_6_): *δ*_H_ = 8.43 (d, *J* = 12 Hz, 2H), 8.29 (d, *J* = 12 Hz, 2H) ppm.

^13^C NMR (100 MHz, DMSO-d_6_): *δ*_C_ = 149.1, 141.2, 131.1, 128.6, 125.0 ppm.

IR (KBr) cm^−1^: 3096, 2850, 1710, 1605, 1550, 1515, 1338, 1286, 1110, 1065, 996, 862, 778, 732, 694, 532, 494, 441.

#### 4-(1*H*-Tetrazol-5-yl)benzonitrile

^1^H NMR (400 MHz, DMSO-d_6_): *δ*_H_ = 8.21 (d, *J* = 12 Hz, 2H), 8.08 (d, *J* = 12 Hz, 2H) ppm.

^13^C NMR (100 MHz, DMSO-d_6_): *δ*_C_ = 138.3, 133.7, 129.4, 128.0, 118.6, 113.7 ppm.

IR (KBr) cm^−1^: 3476, 3092, 3011, 2923, 2861, 2730, 2623, 2231, 1939, 1727, 1622, 1568, 1497, 1432, 1363, 1277, 1205, 1088, 1029, 987, 845, 751, 701, 605, 555.

#### 5-(2-Chlorophenyl)-1*H*-tetrazole

^1^H NMR (300 MHz, DMSO-d_6_): *δ*_H_ = 16.96 (br, 1H), 7.81–7.77 (m, 1H), 7.72–7.67 (m, 1H), 7.65–7.51 (m, 2H) ppm.

#### 5-(4-Bromophenyl)-1*H*-tetrazole

^1^H NMR (300 MHz, DMSO-d_6_): *δ*_H_ = 16.91 (br, 1H), 7.97–7.95 (d, *J* = 8.4 Hz, 2H), 7.83–7.80 (d, *J* = 8.4 Hz, 2H) ppm.

^13^C NMR (100 MHz, DMSO-d_6_): *δ*_C_ = 163.5, 128.3, 124.7, 120.5, 119.4 ppm.

IR (KBr) cm^−1^: 3855, 3435, 3081, 2919, 2855, 2726, 1909, 1721, 1604, 1478, 1429, 1269, 1154, 1061, 991, 873, 830, 740, 598, 500, 453.

#### 5-(4-Chlorophenyl)-1*H*-tetrazole

^1^H NMR (300 MHz, DMSO-d_6_): *δ*_H_ = 8.05–8.02 (d, *J* = 9.9 Hz, 2H), 7.69–7.66 (d, *J* = 9.9 Hz, 2H) ppm.

^13^C NMR (100 MHz, DMSO-d_6_): *δ*_C_ = 150.7, 131.7, 125.4, 124.5, 119.0 ppm.

## 3. Results and discussion

### 3.1. Characterization of the catalyst

Nd-Schiff-base@BMNPs was characterized using the TGA, WDX, SEM, EDS, FT-IR, ICP, and BET techniques.

**Fig. 1:**
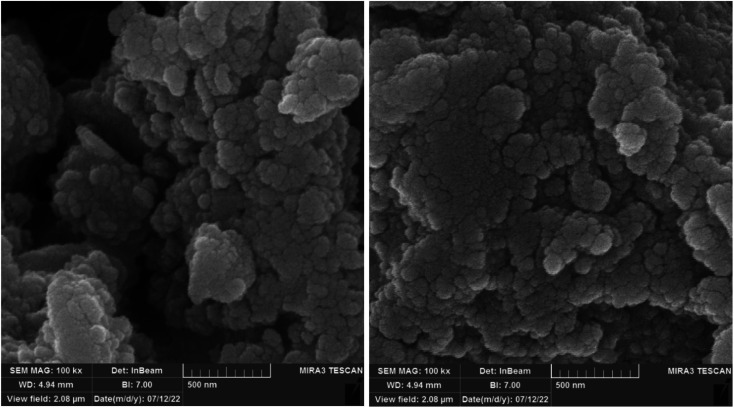
SEM images of Nd-Schiff-base@BMNPs.

A FESEM-TESCAN MIRA III scanning electron microscope (SEM) instrument from Czechia was employed to observe the size and morphology of Nd-Schiff-base@BMNPs particles (Fig. 1). The resulting images from FESEM show that Nd-Schiff-base@BMNPs have uniform spherical shapes and similar diameters in the range of 40–60 nm. Also, an agglomeration was observed in the SEM images of Nd-Schiff-base@BMNPs, which is due to the magnetic property of these nanoparticles.

**Fig. 2:**
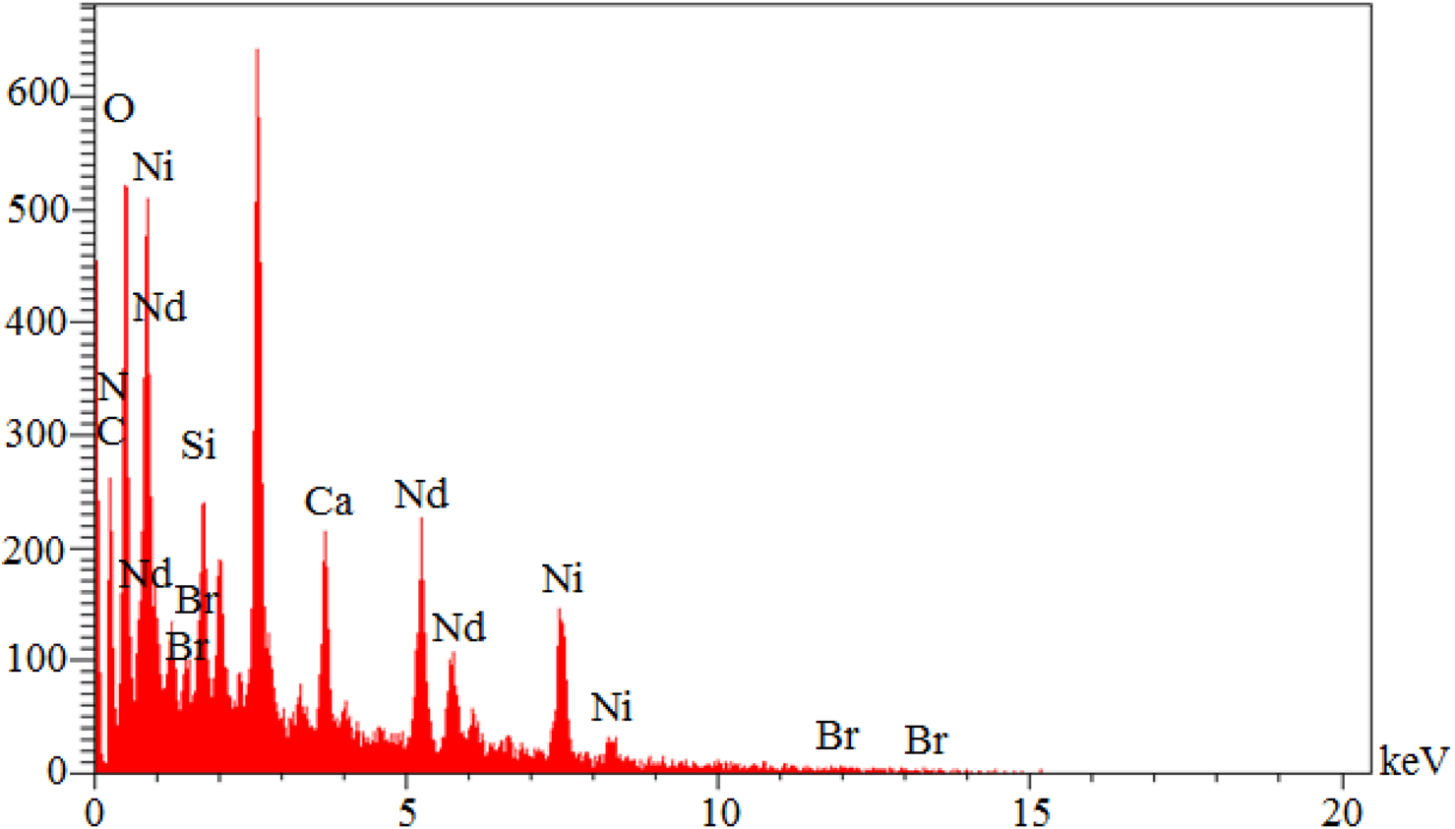
EDS analysis of Nd-Schiff-base@BMNPs.

EDS analysis is a common qualitative method for studying element types. As is known, each element emits specific photons after interaction with X-ray radiation. In EDS analysis, each element is characterized by its specific photon configuration. Also, the peak intensity of each element depends on its concentration. Therefore, an EDS spectrum is a 2-dimensional representation of the special energy (keV) of elements in terms of intensity. Therefore, the elemental organization of Nd-Schiff-base@BMNPs was investigated by EDS analysis (Fig. 2). As indicated, Nd-Schiff-base@BMNPs consist of C, O, Si, N, Br and Nd elements. The content of silica in this catalyst indicates the modification of the biochar magnetic nanoparticle surfaces by CPTMS. The content of nitrogen and bromine in Nd-Schiff-base@BMNPs indicates the immobilization of the Schiff-base ligand on the surface of the biochar magnetic nanoparticles. Also, the content of neodymium indicates that the Nd complex is well supported on the surface of the modified biochar magnetic nanoparticles.

**Fig. 3:**
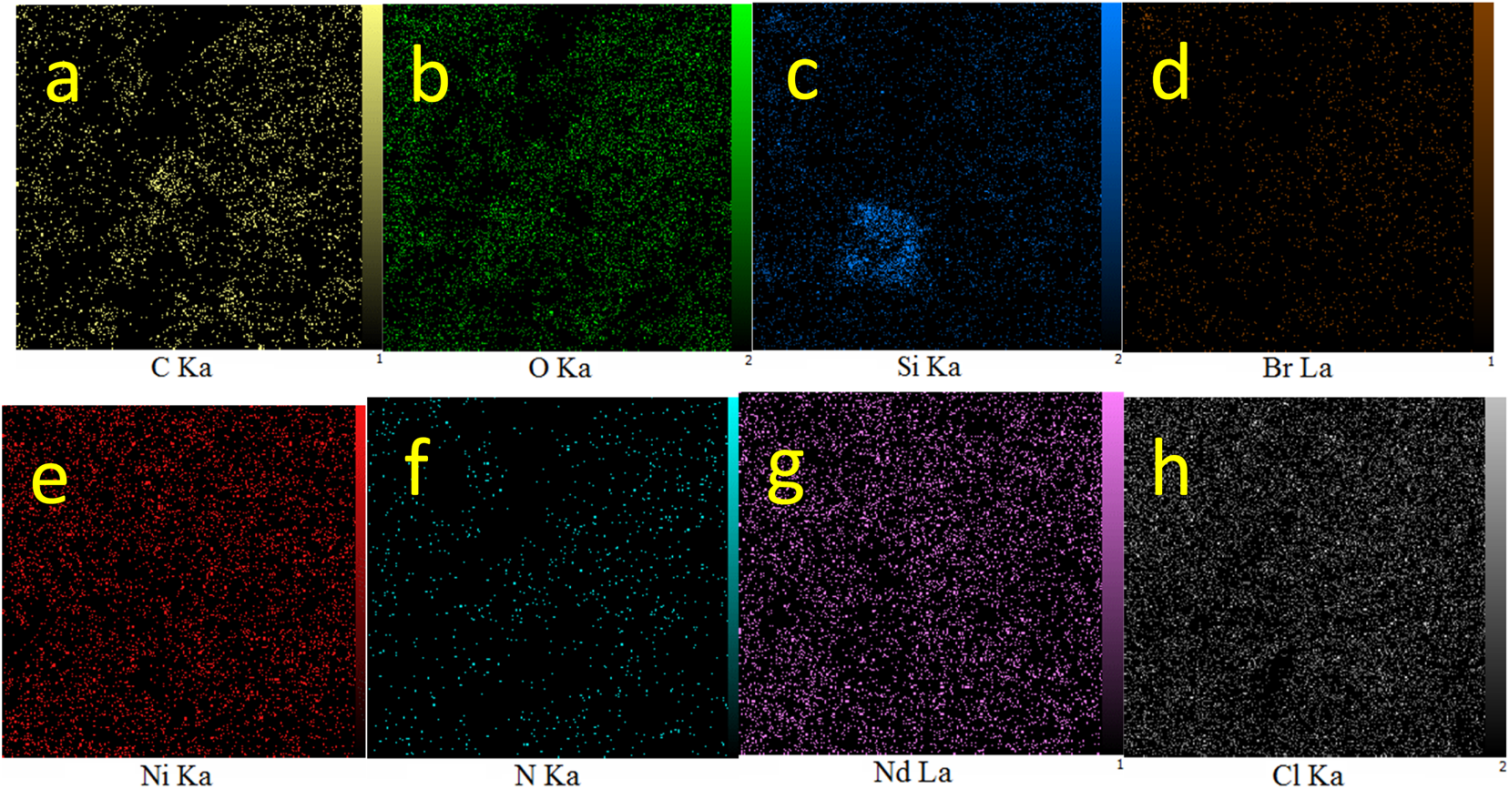
Elemental mapping of (a) C, (b) O, (c) Si, (d) Br, (e) Ni, (f) N, (g) Nd, and (h) Cl for Nd-Schiff-base@BMNPs.

WDX analysis, which is known as elemental mapping, is another qualitative method for studying the distribution and type of elements in a sample. The resulting images from WDX analysis of Nd-Schiff-base@BMNPs are displayed in Fig. 3. The results of WDX analysis indicated the presence of C, O, Si, N, Br and Nd elements, confirming the above results of EDS analysis. Also, the WDX analysis indicates a homogeneous distribution of C, O, Si, N, Br and Nd elements in the structure of Nd-Schiff-base@BMNPs.

Considering that neodymium ions are the catalytic active sites of Nd-Schiff-base@BMNPs, the exact concentration of Nd in this catalyst was calculated by ICP analysis, which was obtained to be 0.2 × 10^−3^ mol g^−1^.

**Fig. 4:**
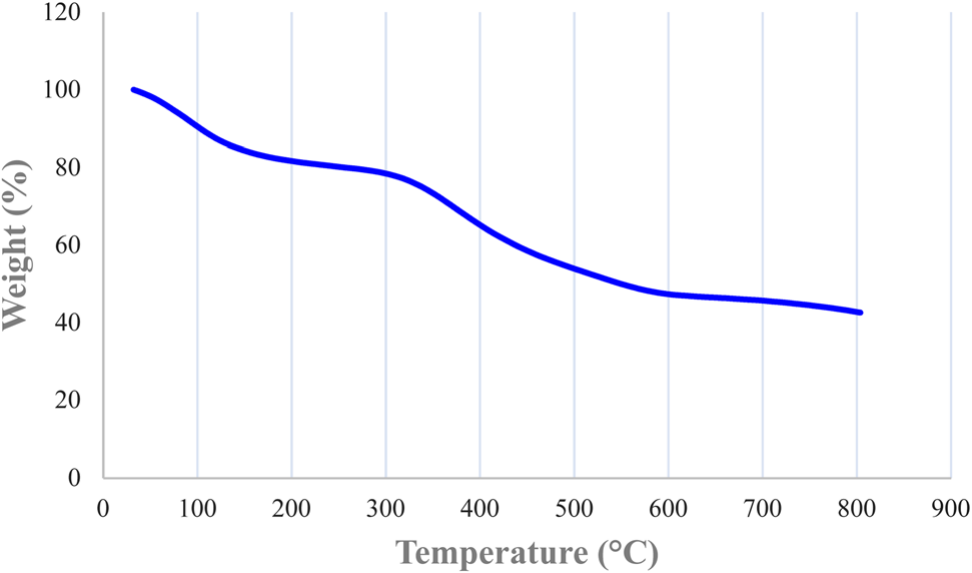
TGA curve of Nd-Schiff-base@BMNPs.

TGA is a useful method for calculating organic layers on solid supports. The approximate content of organic moieties immobilized on the biochar magnetic nanoparticles was studied by TGA. TGA of Nd-Schiff-base@BMNPs was conducted with a NETZSCH STA 449F3 thermal analysis device in the temperature range of 25–800 °C under air atmosphere. The TGA curve of Nd-Schiff-base@BMNPs is shown in Fig. 4. Three steps of weight loss are observed in the TGA curve of Nd-Schiff-base@BMNPs. The absorbent solvents were evaporated below 150 °C, observed as the first step of weight loss (about 20% of total weight). It is important that no weight loss was observed up to 300 °C except solvent evaporation, which means that Nd-Schiff-base@BMNPs are stable and applicable up to 300 °C. The immobilized organic moieties on the biochar magnetic nanoparticles were decomposed above 300–600 °C, observed as the second step of weight loss. This main weight loss indicates the successful immobilization of the Schiff-base ligand on the biochar magnetic nanoparticles, which accounts for approximately 40% of the total weight. Finally, a small weight loss was observed above 600 °C, which may be related to the continuation of biochar pyrolysis.

**Fig. 5:**
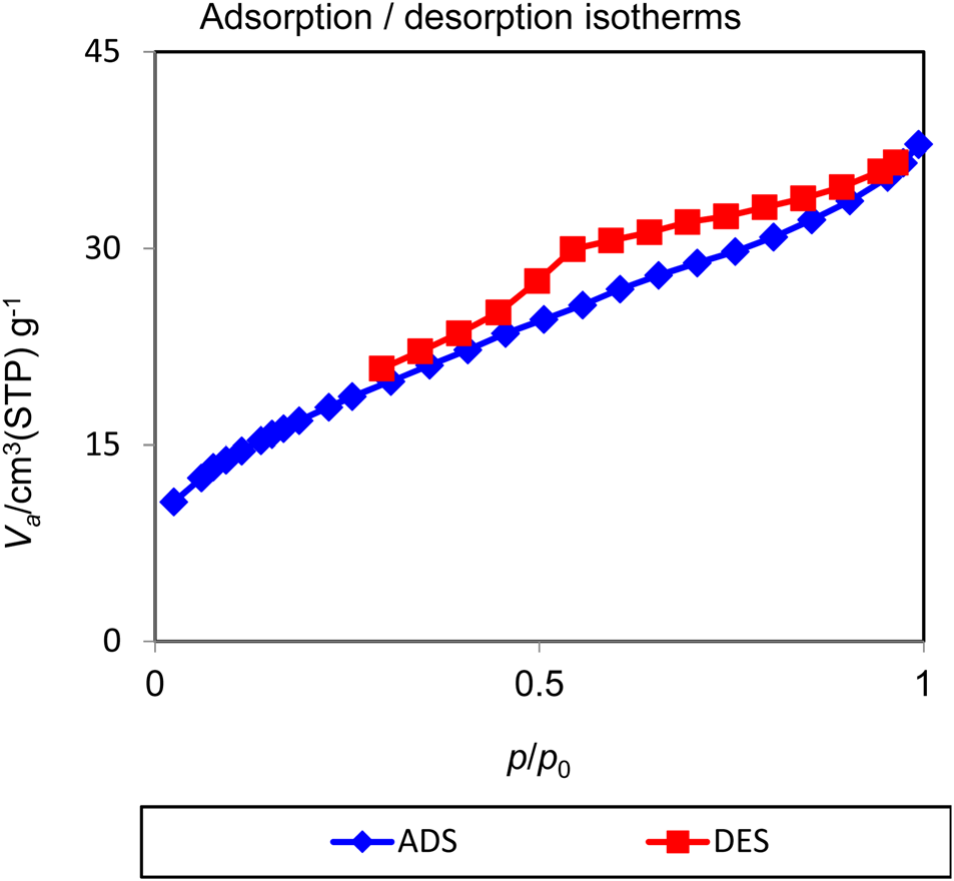
N_2_ adsorption–desorption isotherms of Nd-Schiff-base@BMNPs.

The nitrogen adsorption/desorption technique is a useful tool for calculation of the average pore diameter, surface area and pore volume of porous materials. The resulting isotherms from nitrogen adsorption/desorption are classified in various categories by IUPAC classification. The N_2_ adsorption/desorption isotherms of Nd-Schiff-base@BMNPs were recorded by a standard gas manifold at 77 K with a BELSORP MINI II instrument and then degassed for 2 h at 120 °C with a BEL-PREP-VAC II instrument. The results are displayed in Fig. 5. These isotherms are of the type of isotherm IV in the IUPAC classification, which is in the mesoporous category.^63–66^ Also, N_2_ adsorption/desorption isotherms of Nd-Schiff-base@BMNPs display types of hysteresis loops of H4, which is in the mesoporous carbons category.^65^ Based on the BET method, the specific surface area of Nd-Schiff-base@BMNPs is 16.081 m^2^ g^−1^, which is lower than the surface area of biochar (199–557 m^2^ g^−1^),^50^ as expected. The total pore volume of Nd-Schiff-base@BMNPs is 0.058 cm^3^ g^−1^, also lower than biochar's pore volume (0.22 cm^3^ g^−1^),^50^ indicating the successful immobilization of the Nd-Schiff-base complex on the surface of the biochar magnetic nanoparticles.

**Fig. 6:**
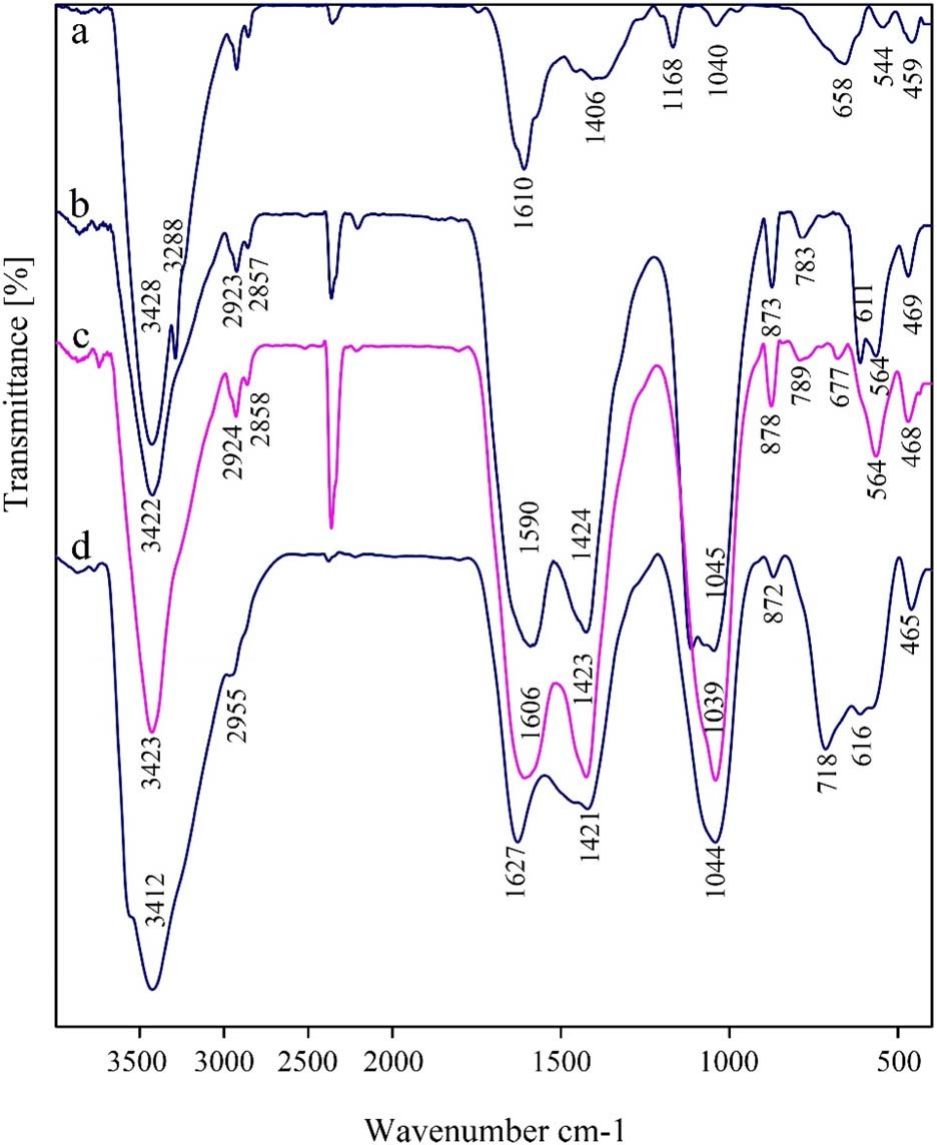
FT-IR spectra for MNiNPs (a), BNPs (b), BMNPs (c) and Nd-Schiff-base@BMNPs (d).

The FT-IR spectra for MNiNPs, BNPs, BMNPs and Nd-Schiff-base@BMNPs were recorded in the solid phase using KBr pellets, which are shown in Fig. 6. The surface of nanoparticles is covered by hydroxyl groups which are characterized by a broad peak above 3400 cm^−1^ in the FT-IR spectra.^36^ Stretching vibration of Ni–O was characterized by a strong band at about 459 cm^−1^ and a band above 610 cm^−1^.^67,68^ The FT-IR spectrum of BNPs showed several characteristic peaks at about 3422 cm^−1^, indicating stretching vibrations of surface oxygen-based functional groups such as alcohol, phenol, and carboxylic acid groups.^45^ Stretching vibrations of C=C bonds were identified by a strong peak at 1590 cm^−1^.^45^ The several bands at 468, 789 and 1039 cm^−1^ correspond to the vibrations of Si–O–Si, indicating successful modification of BMNPs with 3-CPTMS.^26^ Stretching vibrations of C–H bonds were characterized in FT-IR spectra by several peaks lower than 3000 cm^−1^.^36^ Bending vibrations and stretching vibrations of organic layers are observed as peaks in the ranges 600–720 cm^−1^ and 1400–1650 cm^−1^, respectively, which appeared as broad peaks due to the overlap with the peaks of MNiNPs and BNPs. Stretching vibrations of C=N groups were identified by a strong peak at 1627 cm^−1^.^36,45^

### 3.2. Catalytic study of the catalyst

The catalytic performance of Nd-Schiff-base@BMNPs was investigated in the [3 + 2] cycloaddition reaction of NaN_3_ and nitriles toward the homoselective synthesis of heterocyclic tetrazoles. Initially, the reaction conditions for the synthesis of tetrazoles was optimized in the [3 + 2] cycloaddition reaction of NaN_3_ and benzonitrile as a model reaction. First, the effects of the amount of Nd-Schiff-base@BMNPs, solvent and temperature as effective parameters were checked. As seen in entry 1 of Table 1, 5-phenyl-1*H*-tetrazole product was not formed in the absence of Nd-Schiff-base@BMNPs, while the obtained yield of 5-phenyl-1*H*-tetrazole product was increased by increasing the amount of Nd-Schiff-base@BMNPs (Table 1, entries 1–3). Therefore, formation of tetrazoles is dependent on the presence of the catalyst. As seen in entry 3 of Table 1, 50 mg of Nd-Schiff-base@BMNPs was selected as the optimal amount of this catalyst. A lower amount than 50 mg of Nd-Schiff-base@BMNPs is unfavorable for completion of the model reaction (Table 1, entry 2), and higher amounts had no significant effect on reaction time or product yield. In the next step, the effect of the solvent nature was checked, for which polar solvents and non-polar solvents such as H_2_O, PEG-400, DMSO, *n*-hexane and dichloromethane were examined. As seen in Table 1 (entries 4–6) polar solvents provide better conditions for the synthesis of tetrazoles resulting from [3 + 2] cycloaddition reaction of NaN_3_ and nitriles, while non-polar solvents are not suitable for this reaction (Table 1, entries 7 and 8). These results may be due to the solvation of raw materials and products in different solvents. Based on outlined results in Table 1 (entry 3), PEG-400 was selected as a green and safe solvent for optimal conditions. Next, the amount of NaN_3_ was also checked, for which 0.091 g (1.4 mmol) of NaN_3_ per 1 mmol of benzonitrile was selected as the optimal amount of NaN_3_. Increasing the amount of NaN_3_ did not have a significant effect on the reaction time or yield. Finally, the reaction temperature was optimized, the best result being observed at 120 °C (Table 1, entry 3).

**Table 1:** Determination of the best reaction conditions for the synthesis of 5-substituted 1*H*-tetrazoles in the presence of Nd-Schiff-base@BMNPs

Entry	Amount of catalyst (mg)	Solvent	NaN_3_ (mmol)	Time (min)	Temperature (°C)	Yield^a^ (%)
1	—	PEG-400	1.4	180	120	N.R.
2	40	PEG-400	1.4	310	120	89
3	50	PEG-400	1.4	180	120	98
4	50	PEG-400	1.3	180	120	65
5	50	DMSO	1.4	180	120	51
6	50	H_2_O	1.4	180	Reflux	30
7	50	*n*-Hexane	1.4	180	Reflux	Trace
8	50	Dichloromethane	1.4	180	Reflux	Trace
9	50	PEG-400	1.4	180	100	38

a Isolated yield.

**Scheme 4:**
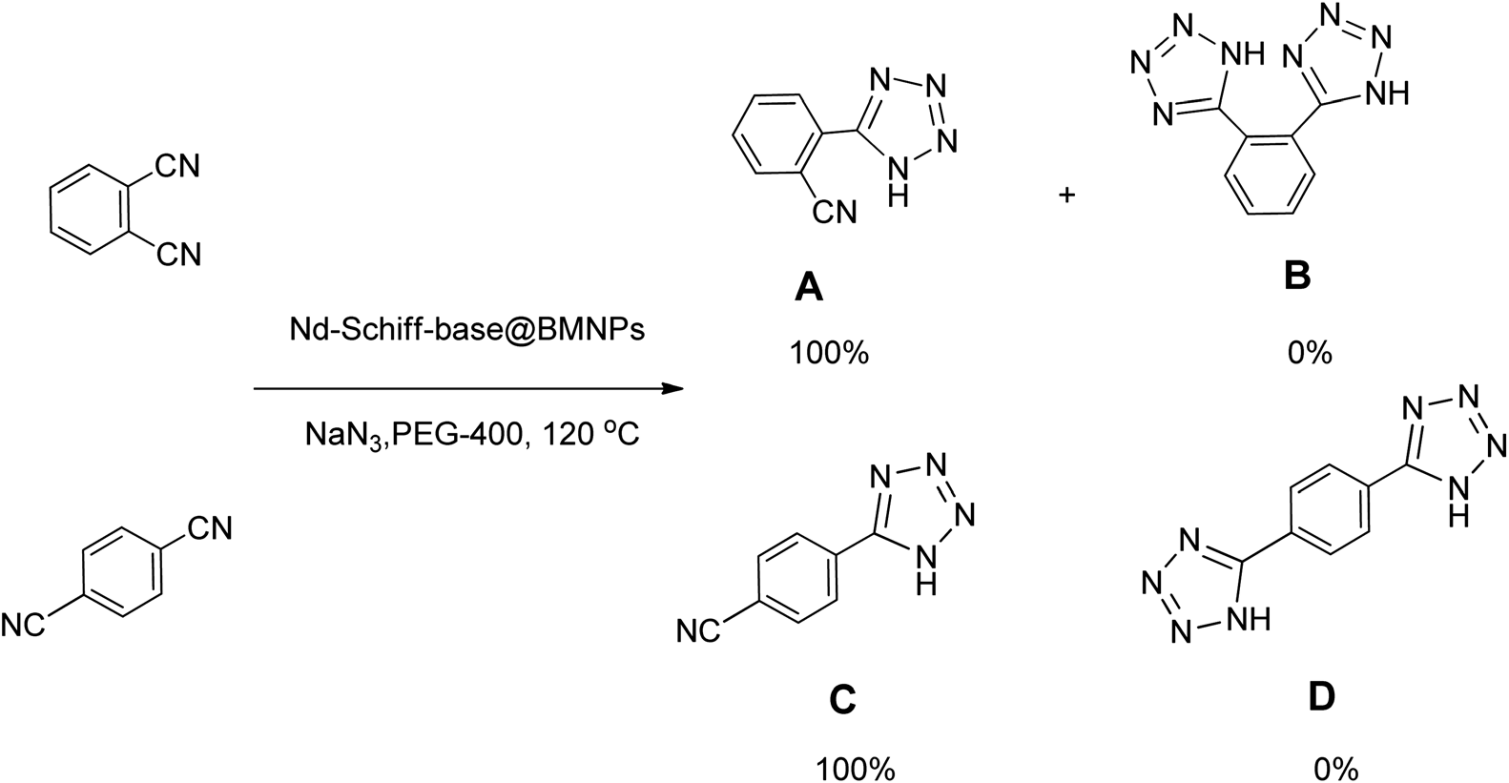
Homoselectivity of Nd-Schiff-base@BMNPs in the [3 + 2] cycloaddition of NaN_3_ with phthalonitrile and terephthalonitrile.

**Fig. 7:**
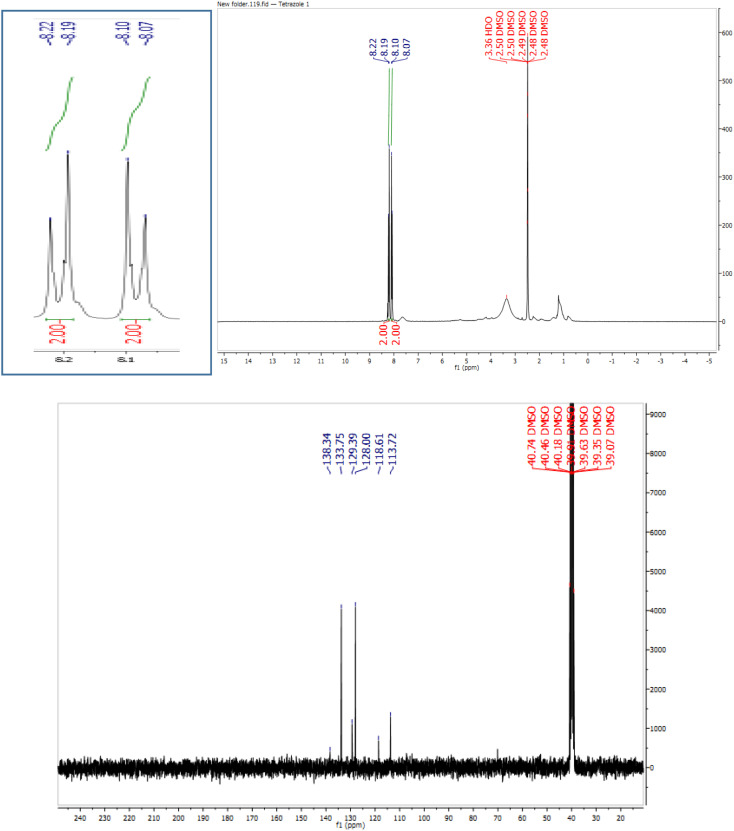
^1^H NMR and ^13^C NMR spectra of 4-(1*H*-tetrazol-5-yl)benzonitrile from [3 + 2] cycloaddition of NaN_3_ with terephthalonitrile.

In continuation, different 5-substituted 1*H*-tetrazole heterocyclic compounds were synthesized through [3 + 2] cycloaddition of NaN_3_ and various benzonitrile derivatives under the above optimized reaction conditions in the presence of Nd-Schiff-base@BMNPs as catalyst. The obtained results are displayed in Table 2. Various benzonitriles as starting materials having electron-withdrawing or -donating substituent groups at the *ortho*, *meta* or *para* position of aromatic ring were examined. In these reactions, final tetrazole products were obtained in good yields within short times in the presence of Nd-Schiff-base@BMNPs as catalyst. More importantly, Nd-Schiff-base@BMNPs exhibit excellent homoselectivity in the formation of heterocyclic tetrazole compounds. In the [3 + 2] cycloaddition reaction of NaN_3_ with dicyano-functionalized benzonitriles (such as phthalonitrile and terephthalonitrile), which have two cyano functional groups in similar positions in their structure, only mono-cycloaddition was observed in the presence of Nd-Schiff-base@BMNPs (Scheme 4). Selectivity and more importantly homoselectivity in organic chemistry, medicinal chemistry and green chemistry are of special importance. The homoselectivity of Nd-Schiff-base@BMNPs in the [3 + 2] cycloaddition reaction of NaN_3_ with phthalonitrile toward the synthesis of 2-(1*H*-tetrazol-5-yl)benzonitrile (A) or 1,2-di(1*H*-tetrazol-5-yl)benzene (B) has been investigated using ^1^H NMR, ^13^C NMR and FT-IR spectroscopies. Also, the homoselectivity of Nd-Schiff-base@BMNPs in the [3 + 2] cycloaddition reaction of NaN_3_ with terephthalonitrile toward the synthesis of 4-(1*H*-tetrazol-5-yl)benzonitrile (C) or 1,4-di(1*H*-tetrazol-5-yl)benzene (D) has been investigated using ^1^H NMR, ^13^C NMR and FT-IR spectroscopies. As mentioned, one of the cyano functional groups selectively participates in the [3 + 2] cycloaddition reaction with NaN_3_ for the synthesis of the corresponding tetrazole, and the other functional group remains unchanged. This remaining cyano functional group is characterized by a sharp peak in the region of 2231 cm^−1^ in the FT-IR spectrum. While if both cyano groups participate in the synthesis of tetrazoles, this peak does not appear in the FT-IR spectrum. Therefore, only products A and C are homoselectively formed in the presence of Nd-Schiff-base@BMNPs (Scheme 4). Also, when terephthalonitrile is used as starting material, if product C is formed, two peaks for aromatic hydrogens should be observed in the ^1^H NMR spectrum. But if product D is formed, only one peak for aromatic hydrogens is observed in the ^1^H NMR spectrum. As can be seen in the ^1^H NMR spectrum of the final synthesized product in the presence of Nd-Schiff-base@BMNPs, two different peaks have been observed for aromatic hydrogens in the region of 8.20 and 8.08 ppm (Fig. 7), so it can be said with certainty that only product C is homoselectively formed in the presence of Nd-Schiff-base@BMNPs. Also, product C shows six peaks in the ^13^C NMR spectrum, but product D shows a maximum of three peaks in the ^13^C NMR spectrum. As seen in the ^13^C NMR spectrum of the product formed in the presence of Nd-Schiff-base@BMNPs, six peaks are seen at 138.3, 133.7, 129.4, 128.0, 118.6 and 113.7 ppm (Fig. 7). Therefore, only product C is homoselectively formed in the presence of Nd-Schiff-base@BMNPs (Scheme 4).

**Table 2:** Synthesis of 5-substituted 1*H*-tetrazoles catalyzed by Nd-Schiff-base@BMNPs

Entry	Nitrile	Product	Time (min)	Yield (%)
1	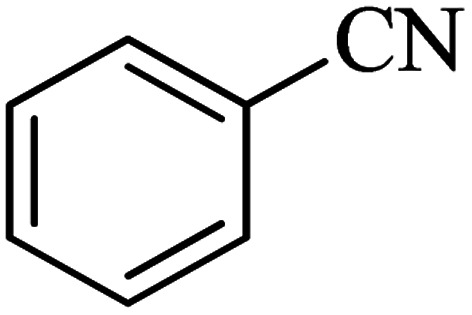	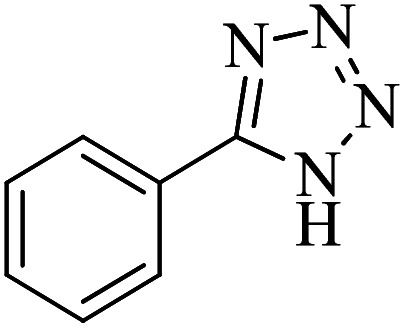	180	98
2	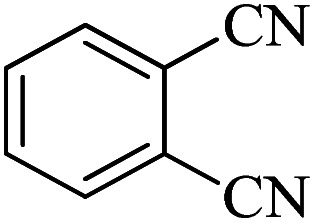	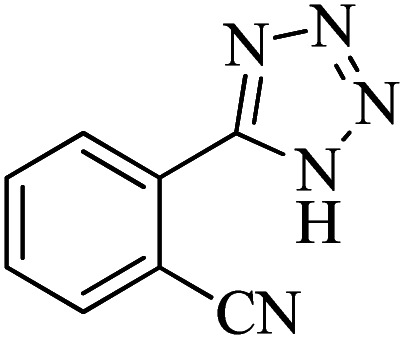	65	94
3	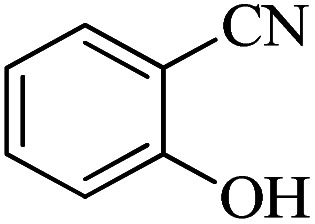	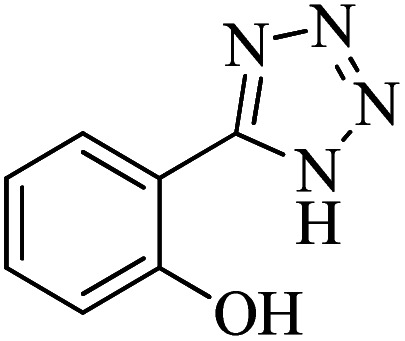	70	95
4	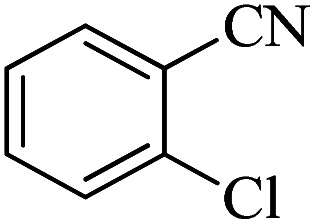	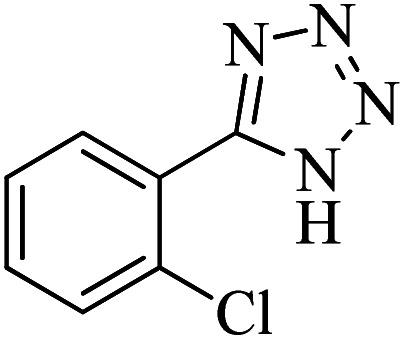	45	93
5	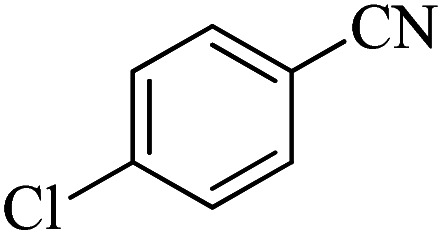	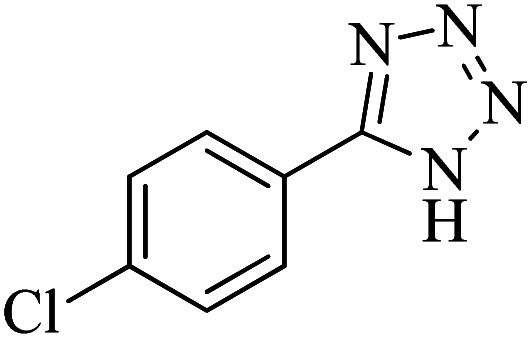	160	92
6	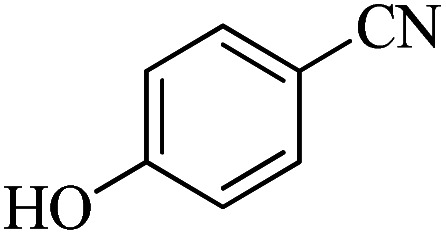	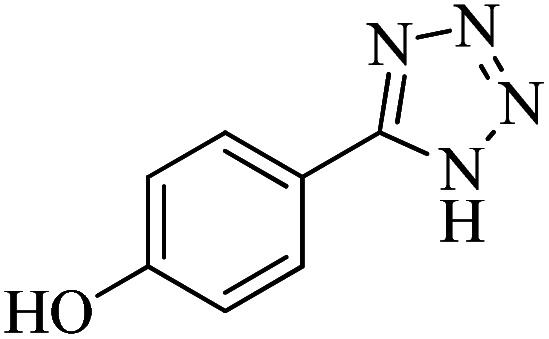	50	93
7	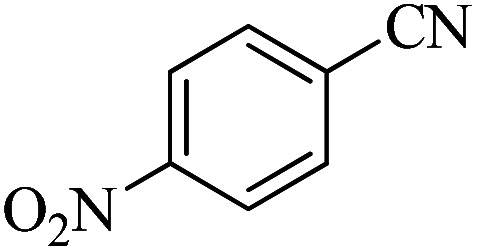	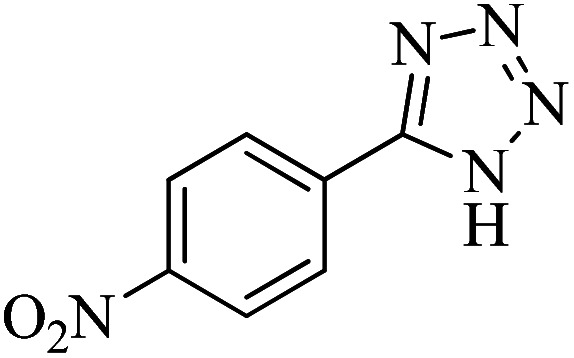	500	90
8	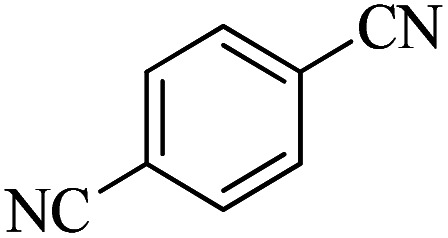	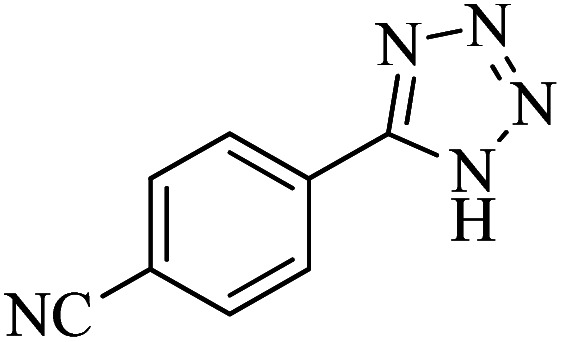	100	96
9	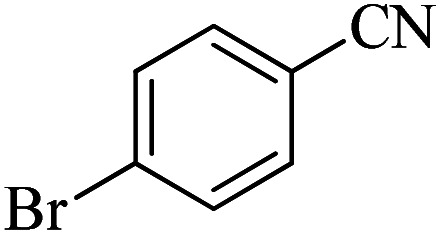	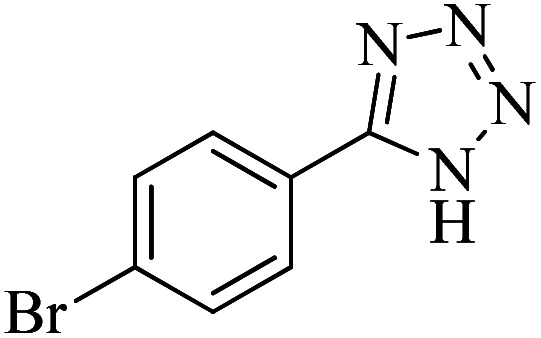	19.5 h	91
10	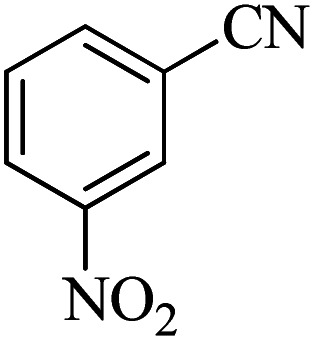	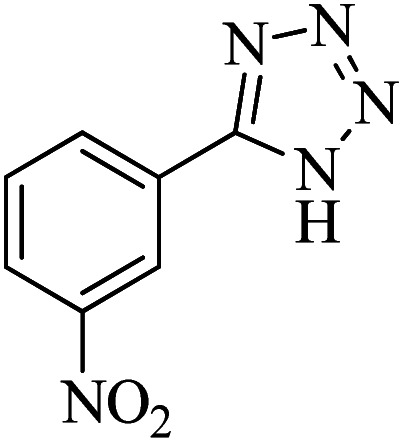	400	89

**Scheme 5:**
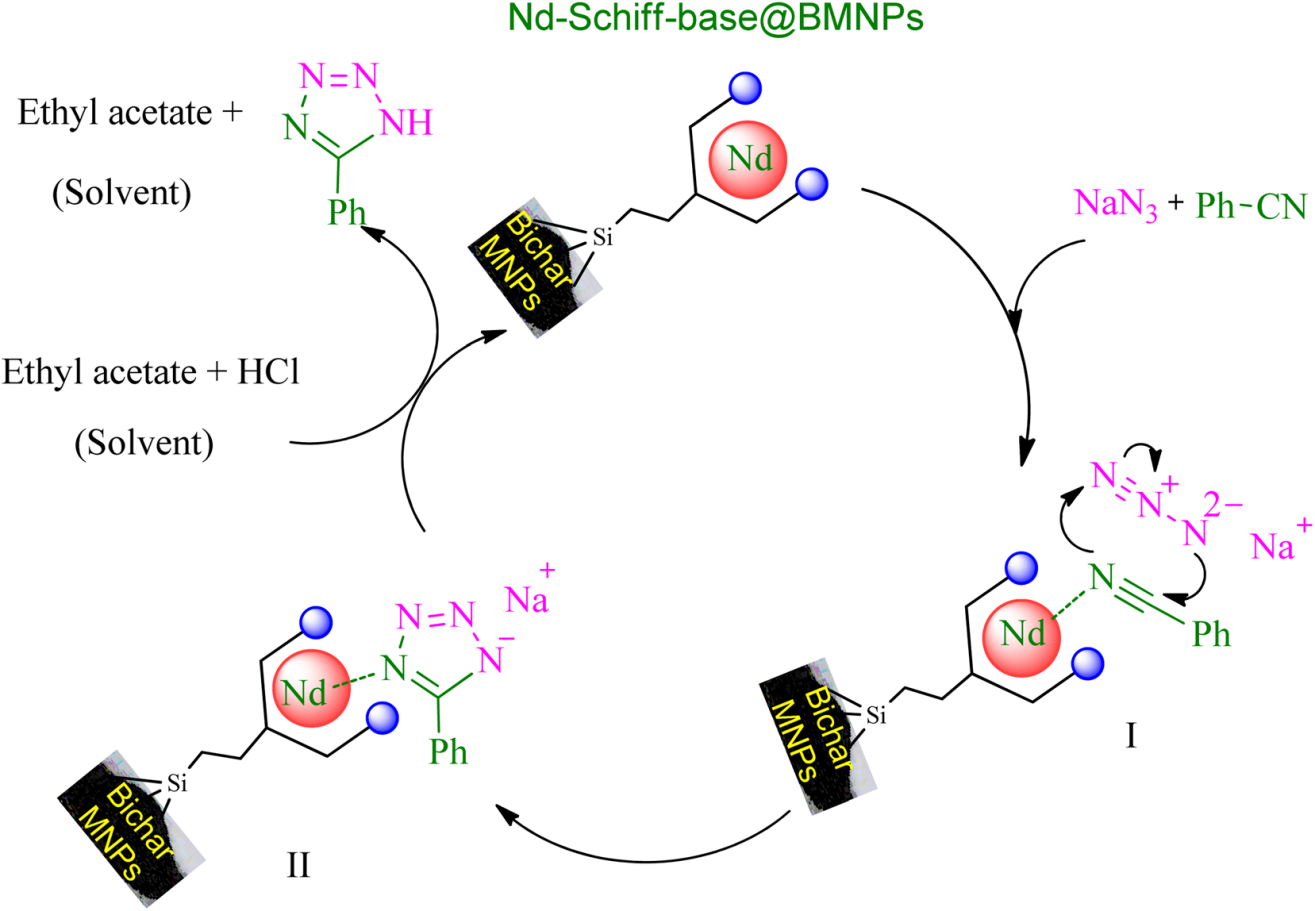
Expected mechanism for the synthesis of tetrazoles in the presence of Nd-Schiff-base@BMNPs.

Based on published articles in the field of catalytic synthesis of tetrazoles,^24,69^ a plausible mechanism is proposed for the synthesis of tetrazoles through [3 + 2] cycloaddition reaction of NaN_3_ and nitriles in the presence of Nd-Schiff-base@BMNPs (Scheme 5). In this suggested mechanism, the interaction of the nitrile functional group with the catalyst causes the nitrile group to become susceptible to nucleophilic attack, which leads to the formation of intermediate II through the [3 + 2] cycloaddition reaction with azide ions. In this stage, intermediate II is formed as a sodium salt form of target tetrazoles. Intermediate II is an ionic compound that is better solvated in protic polar solvents. Therefore, as shown in Table 1, protic polar solvents provide better conditions for the synthesis of tetrazoles resulting from [3 + 2] cycloaddition reaction of NaN_3_ and nitriles, while non-polar solvents are not suitable for this reaction. Therefore, to convert the salt form (intermediate II) into a final molecular tetrazole compound, HCl was added in the workup step, in which final tetrazoles were extracted in ethyl acetate and the regenerated catalyst is returned for another catalytic cycle.

### 3.3. Reusability of the catalyst

**Fig. 8:**
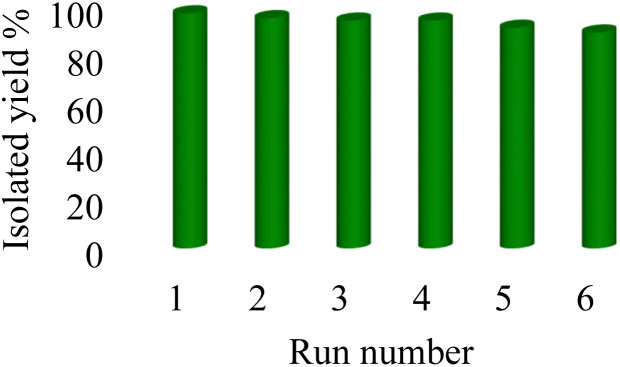
The recoverability and reusability of Nd-Schiff-base@BMNPs in the synthesis of 5-phenyl-1*H*-tetrazole.

Stability, reusability, biocompatibility, selectivity, practicability and availability are the important, main, and determinative factors of different catalysts that are emphasized by principles of green chemistry. Therefore, green chemistry emphasizes the use of reusable heterogeneous catalysts due to their being economical, reusable, and environmentally friendly.^70–76^ Therefore, to examine the reusability of Nd-Schiff-base@BMNPs, [3 + 2] cycloaddition reaction of NaN_3_ with benzonitrile toward the synthesis of 5-phenyl-1*H*-tetrazole was selected under the optimized conditions in Table 1. In this regard, Nd-Schiff-base@BMNPs catalyst was recovered by an external magnet after completion of the reaction and then it was used again without any activation in the next run. As shown in Fig. 8, Nd-Schiff-base@BMNPs can be recovered and recycled up to 6 times without significant reduction in the catalytic practicability.

**Fig. 9:**
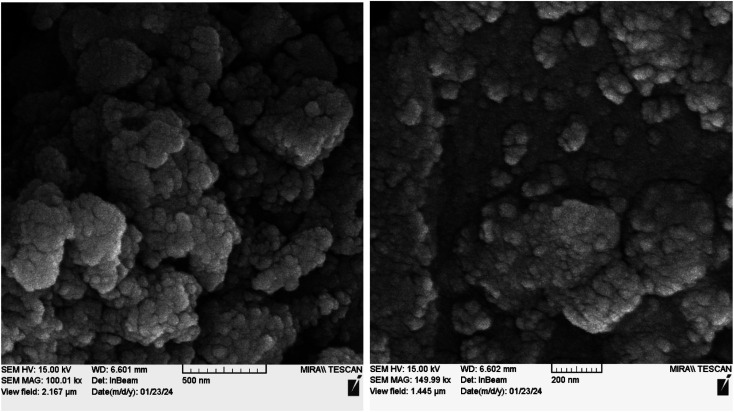
SEM images of recovered Nd-Schiff-base@BMNPs.

The morphology and particle size of recovered Nd-Schiff-base@BMNPs were studied by an electron microscope (Model MIRA3 TESCAN-XMU). The SEM images of recovered Nd-Schiff-base@BMNPs after reuse are shown in Fig. 9. As displayed in Fig. 9 no significant change was observed in morphology and particle size after being reused, based on the SEM results. SEM images of recovered Nd-Schiff-base@BMNPs showed a good similarity with SEM images of fresh catalyst before reuse. Therefore Nd-Schiff-base@BMNPs are stable under reaction conditions for synthesis of tetrazoles.

### 3.4. Comparison of the catalyst

The practicability of Nd-Schiff-base@BMNPs in comparison with other reported catalysts is illustrated in Table 3. In this table, the [3 + 2] cycloaddition reaction of NaN_3_ and unfunctionalized benzonitrile in the presence of Nd-Schiff-base@BMNPs is compared with that using other reported catalysts. As indicated, Nd-Schiff-base@BMNPs provided 98% of product (5-phenyl-1*H*-tetrazole) after 3 h, being superior to the other catalysts in terms of product yields and reaction times. Also, most of the other catalysts are limited by long reaction times, utilize hazardous solvents, involve time-consuming reactions or difficulty in recovery of the catalysts, use unrenewable materials and utilize chemical materials for synthesis of the catalyst support. In contrast, the formation of tetrazoles in the presence of Nd-Schiff-base@BMNPs as a reusable nanocatalyst was rapid in a green solvent (PEG-400) with excellent yield. Also, biochar is synthesized from renewable materials as a best way for waste recycling. In addition, Nd-Schiff-base@BMNPs can be magnetically recovered and reused.

**Table 3:** Comparison results of Nd-Schiff-base@BMNPs with other catalysts for synthesis of 5-phenyl-1*H*-tetrazole

Entry	Catalyst	Time (h)	Yield (%)	Ref.
1	CoY zeolite	14	90	77
2	Cu–Zn alloy nanopowder	10	95	78
3	B(C_6_F_5_)_3_	8	94	79
4	Fe_3_O_4_@SiO_2_/salen Cu(ii)	7	90	80
5	Fe_3_O_4_/ZnS HNSs	24	81.1	81
6	Mesoporous ZnS	36	86	82
7	AgNO_3_	5	83	83
8	CuFe_2_O_4_	12	82	84
9	Nano ZnO/Co_3_O_4_	12	90	85
10	Ni–MP(AMP)_2_@Fe–biochar	3.8	92	47
11	Fe_3_O_4_@boehmite NPs	4	97	60
12	Nd-Schiff-base@BMNPs	3	98	This work

## 4. Conclusion

As is known, reusable and selective catalyst species, inexpensive materials, available and safe solvents, atom-economic processes, renewable materials and waste recycling are very important principles in green chemistry and industrial processes. Therefore in this work, magnetic biochar NPs were formed from pyrolysis of chicken manure (as a renewable material) as a new procedure for waste recycling. In order to improve its recovery from a reaction mixture, the synthesized biochar was magnetized using magnetic nickel nanoparticles. Then, it was used as an inexpensive, available and novel support for immobilization of neodymium Schiff-base complex as a selective, reusable, available, stable, and practicable catalyst. This nanocatalyst (Nd-Schiff-base@BMNPs) was characterized by TGA, WDX, SEM, EDS, FT-IR, ICP, and BET techniques. Then, the catalytic performance of Nd-Schiff-base@BMNPs was investigated in the homoselective synthesis of tetrazole derivatives in PEG-400 as a green, safe and available solvent. All tetrazole products were synthesized with high atom economy in excellent yields, which points to the high efficiency and practicability of Nd-Schiff-base@BMNPs as catalyst. Nd-Schiff-base@BMNPs were recovered and recycled for six runs without metal leaching or significant reduction of their activity, which is indicative of the stability and reusability of this catalyst. Also, Nd-Schiff-base@BMNPs provide an excellent selectivity in the synthesis of tetrazole derivatives.

## Data availability

Data available in main article file and ESI.†

## Author contributions

Bahman Tahmasbi: supervision, writing – original draft, writing review & editing. Parisa Moradi: data collection, methodology. Mitra Darabi: data collection, methodology.

## Conflicts of interest

Authors declare no conflict of interest or competing interests.

## Supplementary Material

NA-006-D3NA01087B-s001
