# Supplementary material for: A new neodymium complex on renewable magnetic biochar nanoparticles as an environmentally friendly, recyclable and efficient nanocatalyst in the homoselective synthesis of tetrazoles
Source: Nanoscale Adv. 2024 Feb 26;6(7):1932–44. doi: 10.1039/d3na01087b (PMC10964755; doi:10.1039/d3na01087b)

Electronic Supplementary Material (ESI) for Nanoscale Advances.
This journal is © The Royal Society of Chemistry 2024

A new neodymium complex on renewable magnetic biochar nanoparticles as an environmentally, recyclable and efficient nanocatalyst in the homo-selective synthesis of tetrazoles

Bahman Tahmasbi*, Parisa Moradi, Mitra Darabi

Department of Chemistry, Faculty of Science, Ilam University, P. O. Box69315516, Ilam, Iran.

Abstract

Inexpensive, stable, selective, and recyclable nanocatalysts, waste regenerating, utilize safe and available solvents are interest and important factors in laboratory science and industrial applications of green chemistry. Therefore herein, biochar nanoparticles (BNPs) were synthesized through chicken manure pyrolysis as a novel method for waste recycling. Then, in order to improve its recyclability, the obtained BNPs were magnetized using magnetic nickel nanoparticles. Then, the surface of biochar magnetic nanoparticles (BMNPs) was modified by (3-chloropropyl) trimethoxysilane (3-CPTMS) and further a novel neodymium Schiff-base complex was immobilized on the surface of modified BMNPs, which labelled as Nd-Schiff-base@BMNPs. Obtained supported neodymium complex was used as practical, selective, biocompatibility, commercial, and reusable heterogeneous nanocatalyst. The biochar support of this nanocatalyst was formed from pyrolysis of chicken manure, therefore, it is cheap, economically viable, green and also compatible with the principles of green chemistry. Nd-Schiff-base@BMNPs acts selectively in organic reactions and also it can easily recovered using external magnet and reused, which is compatible with the principles of green chemistry. This nanocatalyst was characterized by wavelength dispersive X-ray spectroscopy (WDX), scanning electron microscopy (SEM), energy-dispersive X-ray spectroscopy (EDS), thermogravimetric analysis (TGA), Fourier Transform Infrared Spectroscopy (FT-IR), Inductively Coupled Plasma (ICP), and N_2 adsorption-desorption (BET method) techniques. At the next step, the catalytic utilize of Nd-Schiff-base@BMNPs was investigated in the homoselective synthesis of 5-substituted-1H-tetrazole compounds from [3+2] cycloaddition of sodium azide (NaN_3) and organo-nitriles in PEG-400 as a green solvent. Utilize PEG-400 solvent offers various advantages e.g. cheapness, readily available, and environmentally friendly solvent and as well as rapid separation and high purity of products. Therefore, this work is fully compatible with the principles of green chemistry.

*Address correspondence to Department of Chemistry, Faculty of Science, Ilam University, P. O. Box 69315516, Ilam, Iran. E-mail address of B. Tahmasbi: b.tahmasbi@ilam.ac.ir, bah.tahmasbi@gmail.com

^{1}H NMR spectral data

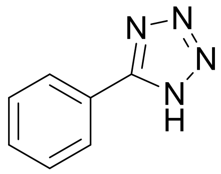

5-phenyl-1H-tetrazole

^{1}H NMR (300 MHz, DMSO-d6): δ_H= 8.04-8.01 (m, 2H), 7.63-7.58 (m, 3H) ppm.

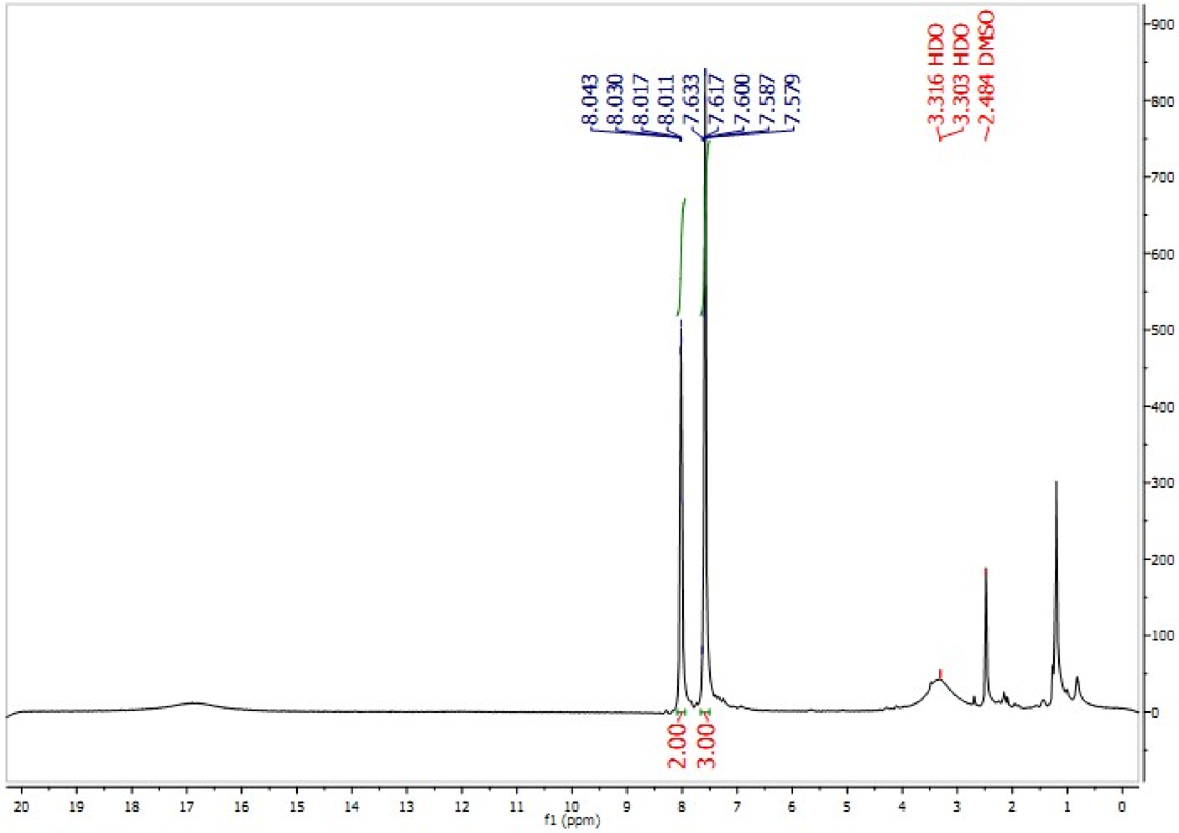

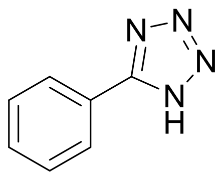

5-phenyl-1H-tetrazole

^{13}C NMR (100 MHz, DMSO-d6): δ_C= 165.7, 131.6, 129.8, 127.3, 124.5 ppm.

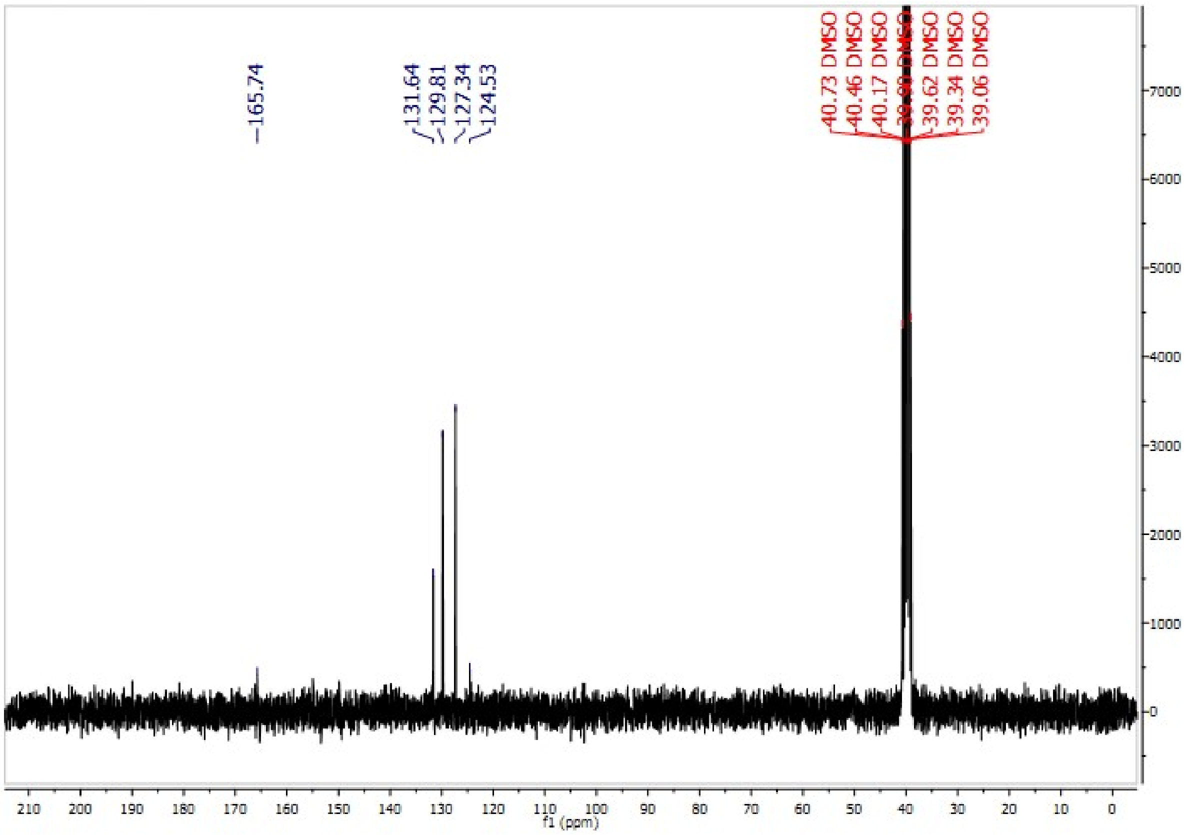

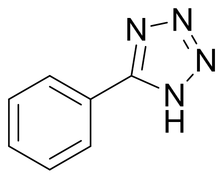

5-phenyl-1H-tetrazole

IR (KBr) cm^{-1}: 3426, 3128, 3056, 2917, 2864, 2701, 2607, 1852, 1735, 1606, 1559, 1472, 1405, 1248, 1153, 1089, 986, 954, 784, 718, 690, 491.

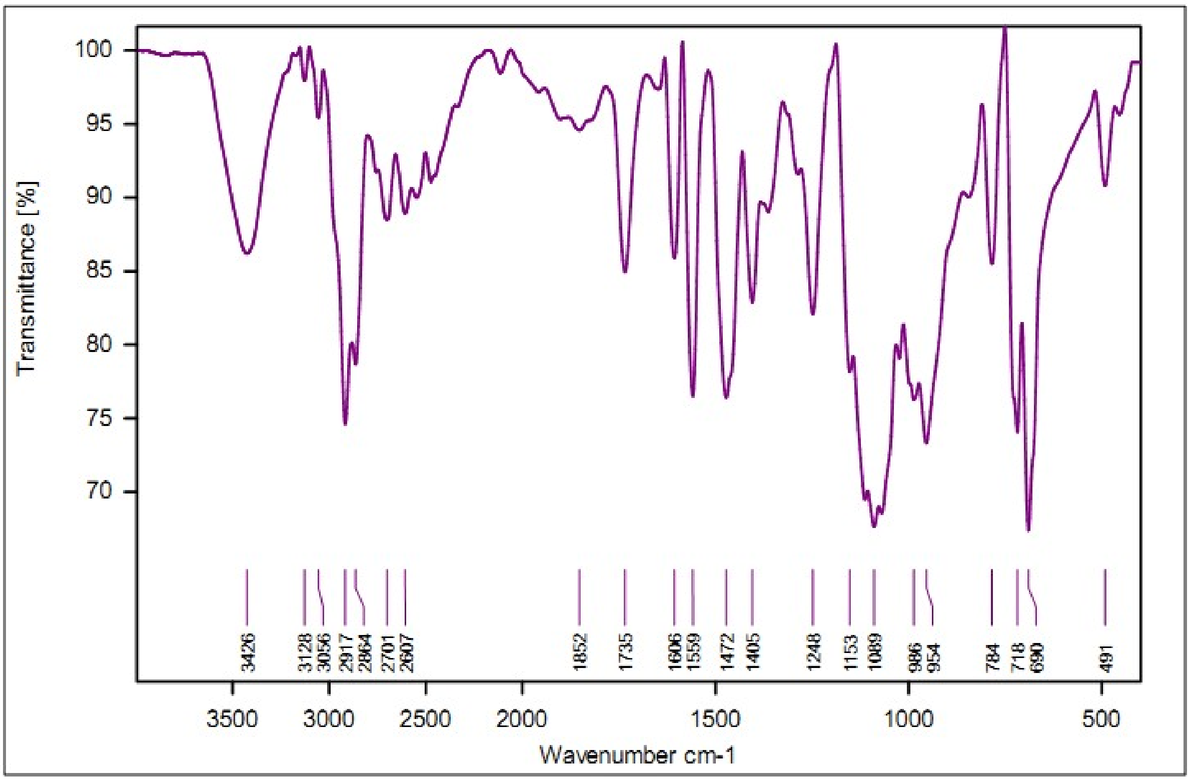

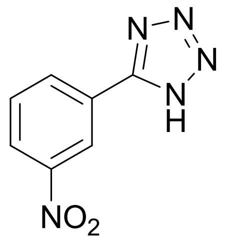

5-(3-nitrophenyl)-1H-tetrazole

^{1}H NMR (300 MHz, DMSO-d6): δ_H= 8.78 (s, 1H), 8.44-8.41 (d, *J*= 7.8 Hz, 1H), 8.39-8.36 (d, *J*= 8.1 Hz, 1H), 7.89-7.84 (t, *J*= 8.1 Hz, 1H) ppm.

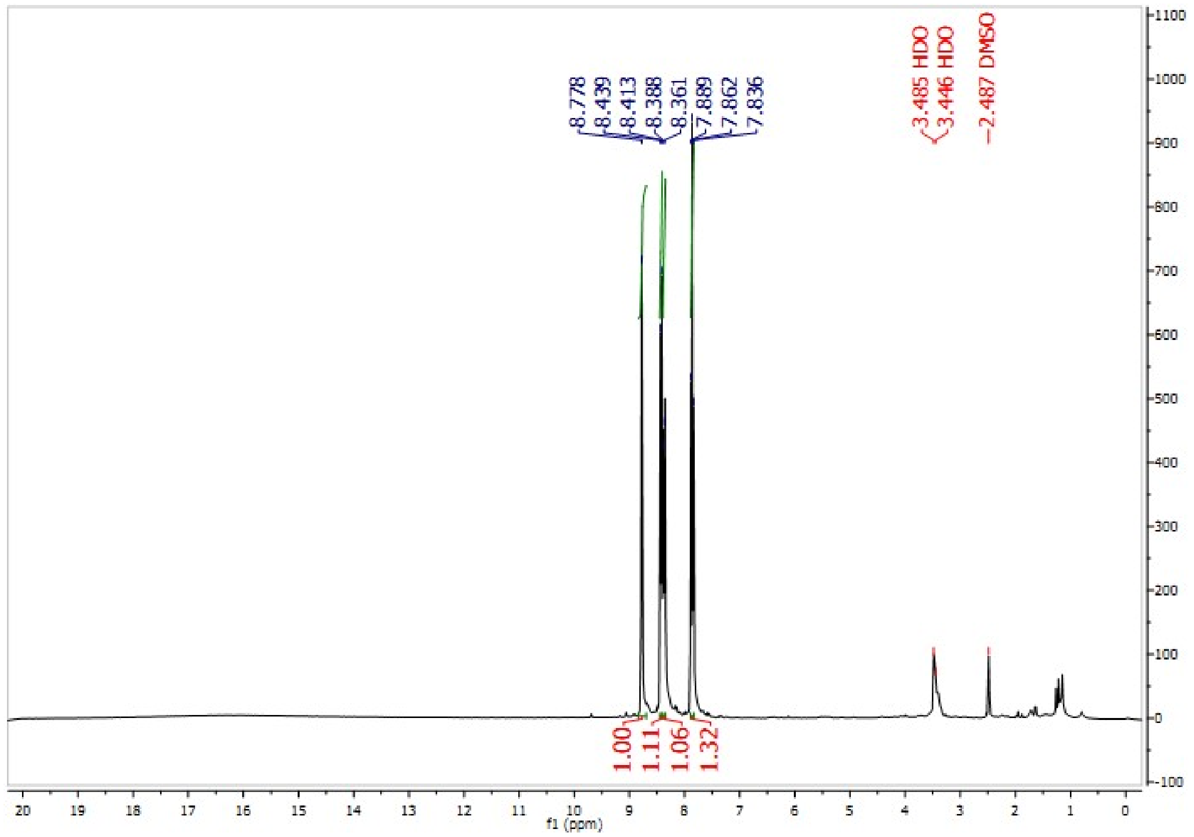

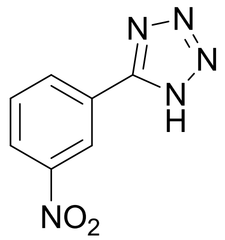

5-(3-nitrophenyl)-1H-tetrazole

^{13}C NMR (100 MHz, DMSO-d6): δ_C= 155.3, 148.6, 133.4, 131.5, 126.5, 125.9, 121.8 ppm.

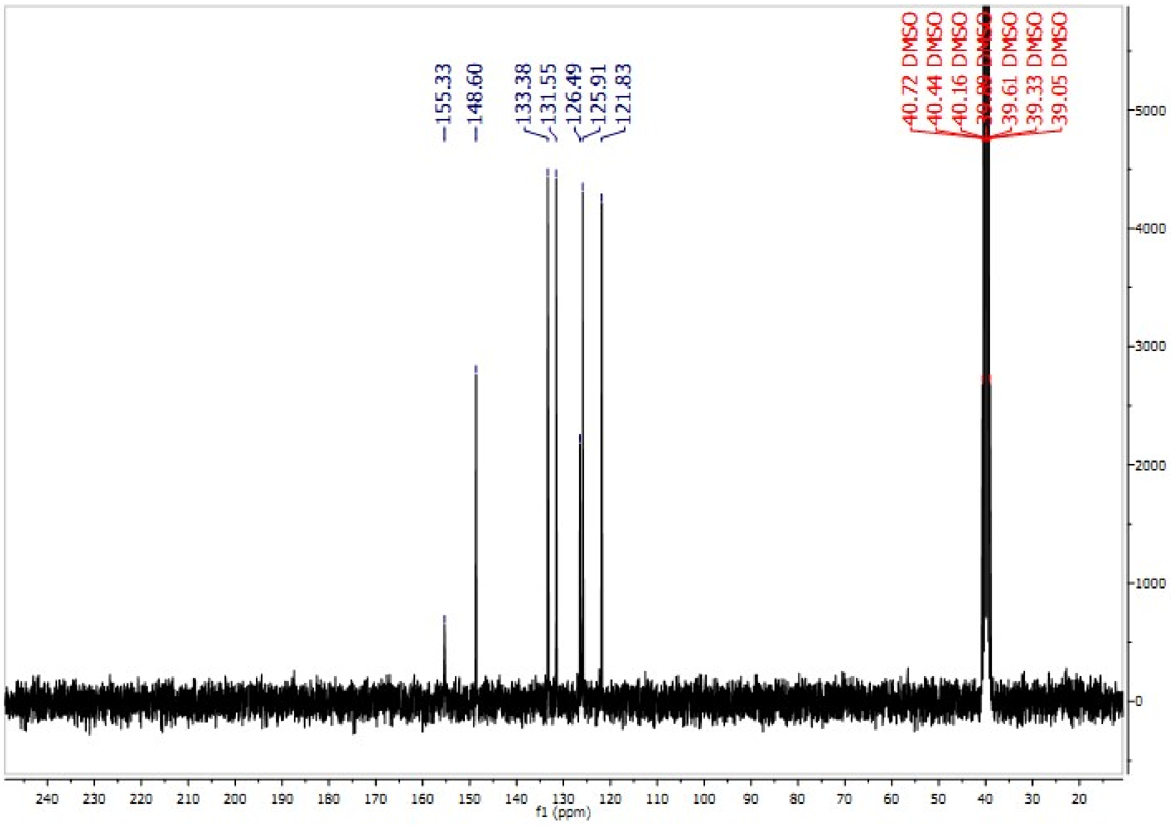

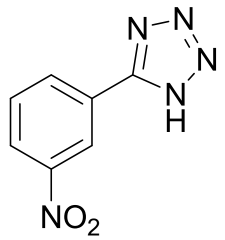

5-(3-nitrophenyl)-1H-tetrazole

IR (KBr) cm^{-1}: 3451, 3306, 3088, 2897, 2753, 1620, 1526, 1347, 1157, 1076, 1013, 913, 866, 820, 735, 452.

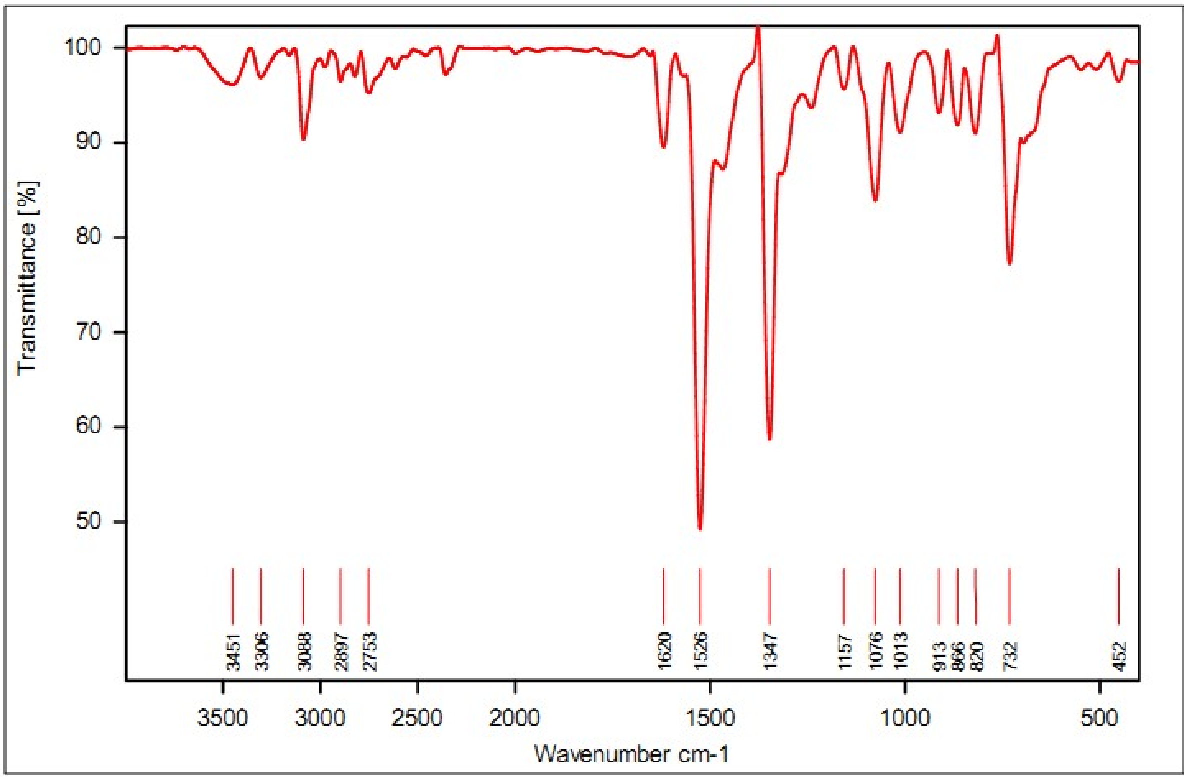

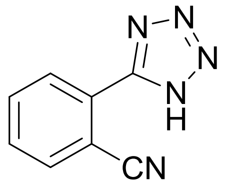

2-(1H-tetrazol-5-yl)benzonitrile

^{1}H NMR (300 MHz, DMSO-d6): δ_H= 8.09-8.05 (t, *J*= 7.2 Hz, 2H), 7.94-7.89 (t, *J*= 7.8 Hz, 1H), 7.79-7.74 (t, *J*= 7.8 Hz, 1H) ppm.

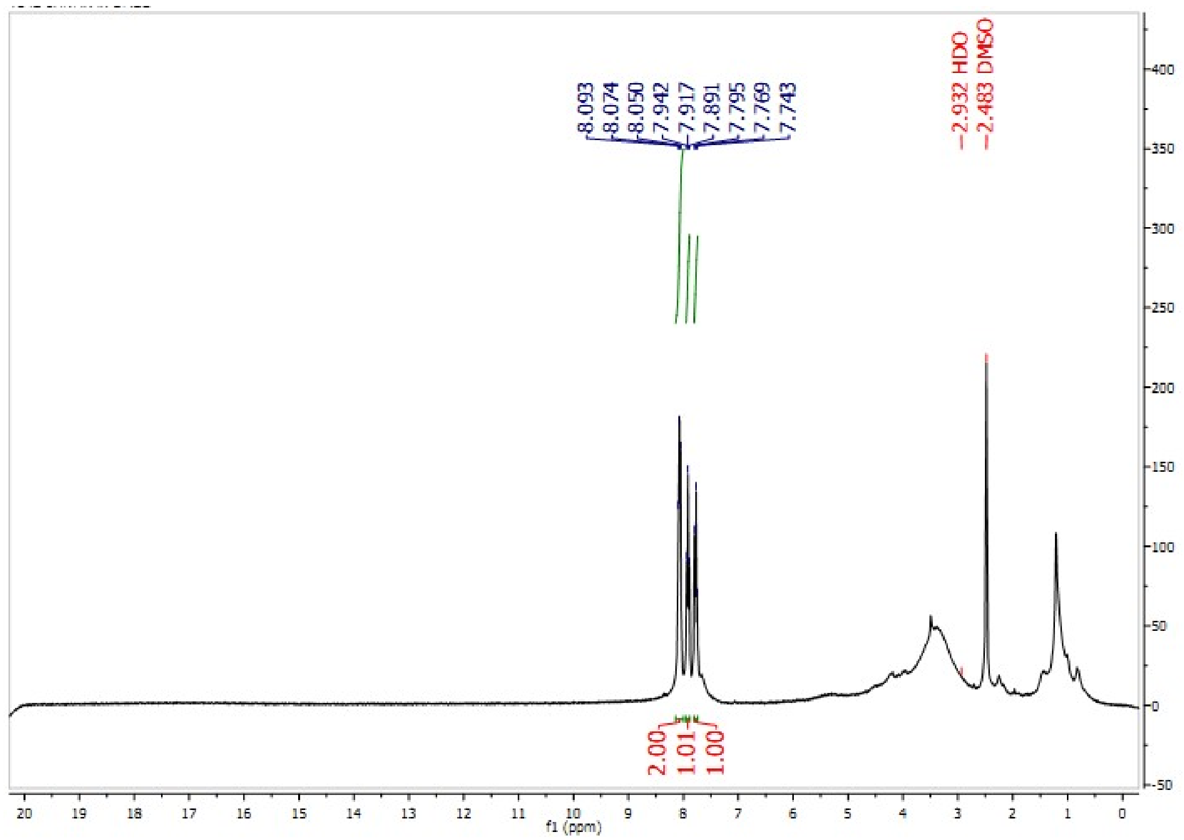

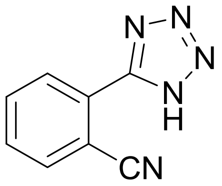

2-(1H-tetrazol-5-yl)benzonitrile

IR (KBr) cm^{-1}: 3397, 2917, 2228, 1732, 1573, 1458, 1357, 1245, 1106, 949, 843, 775, 510.

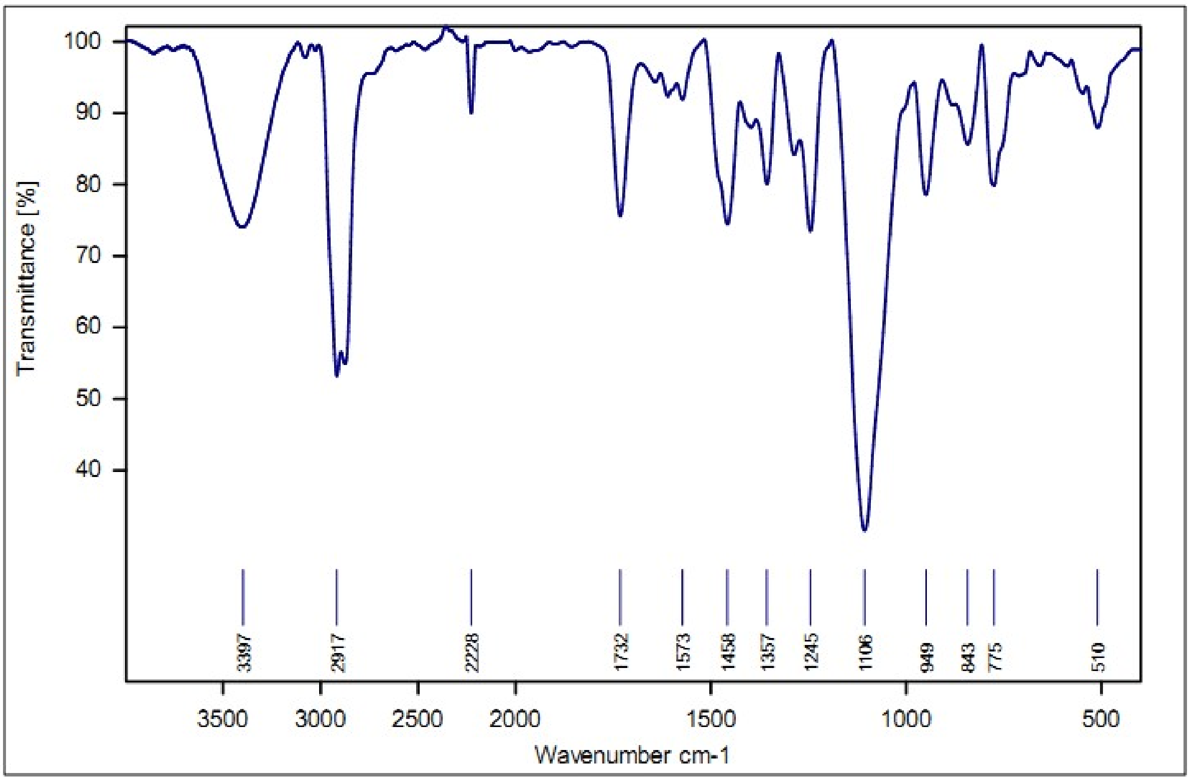

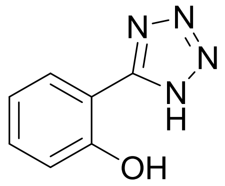

2-(1H-tetrazol-5-yl)phenol

^{1}H NMR (300 MHz, DMSO-d6): δ_H= 7.99-7.96 (d, *J*= 7.8 Hz, 1H), 7.41-7.37 (t, *J*= 7.5 Hz, 1H), 7.07-7.04 (d, *J*= 8.4 Hz, 1H), 7.01-6.96 (t, *J*= 7.2 Hz, 1H), 3.37 (br, 1H) ppm.

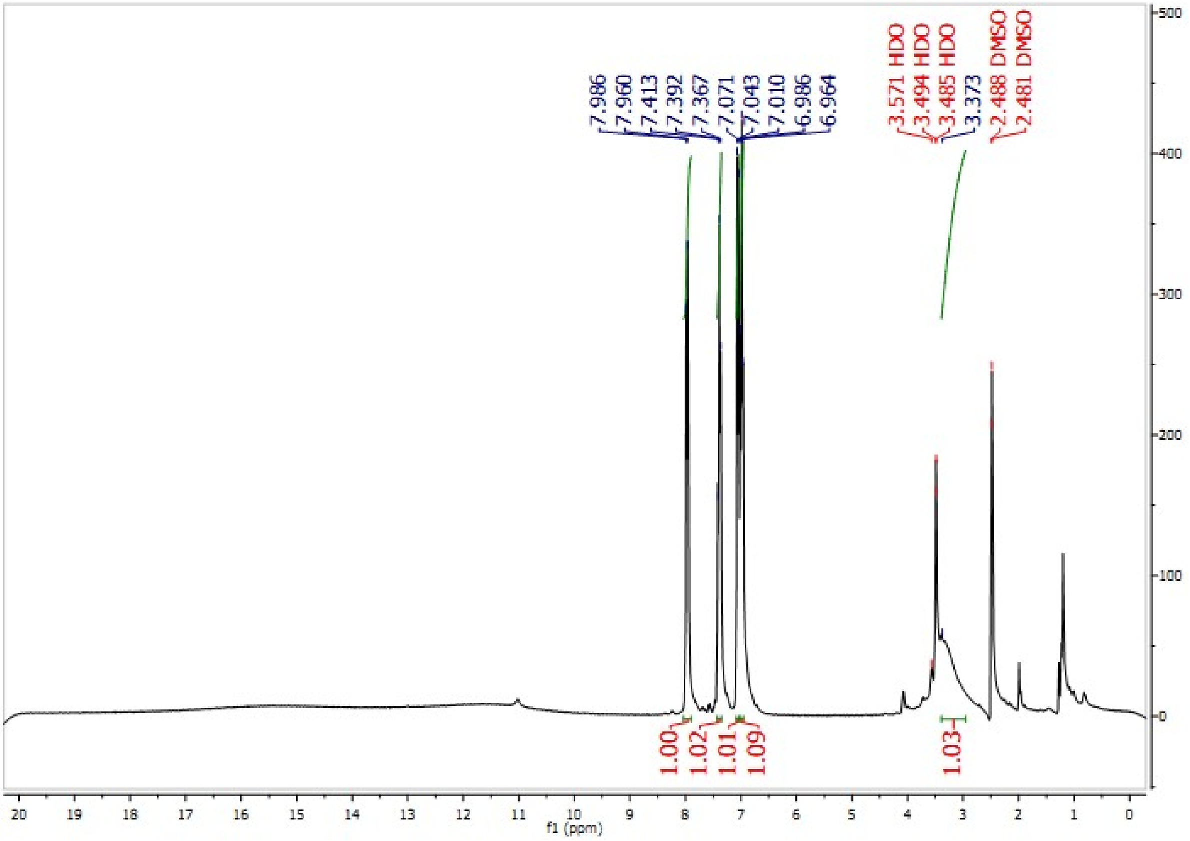

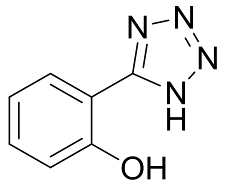

2-(1H-tetrazol-5-yl)phenol

^{13}C NMR (100 MHz, DMSO-d6): δ_C= 155.7, 152.0, 133.0, 129.4, 120.1, 116.7, 110.9 ppm.

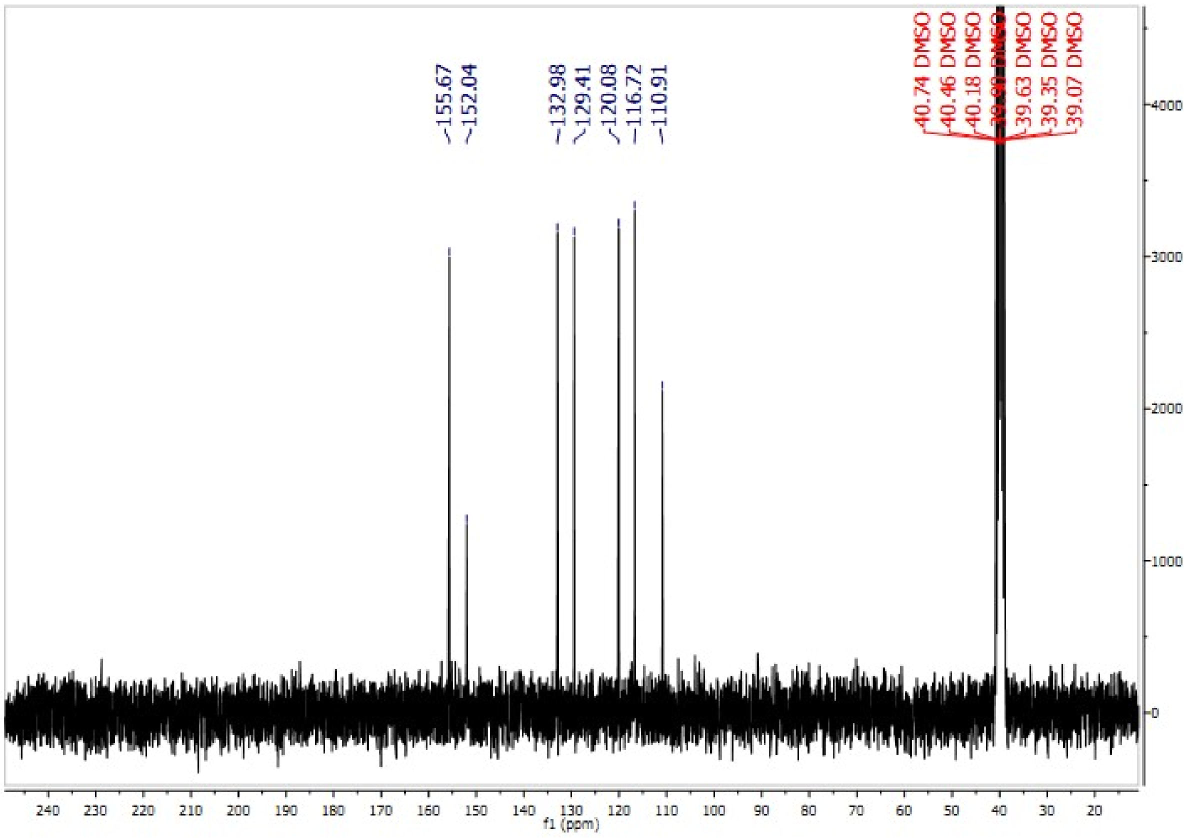

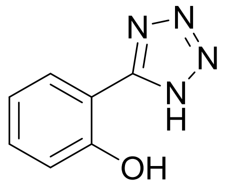

2-(1H-tetrazol-5-yl)phenol

IR (KBr) cm^{-1}: 3446, 3254, 3060, 2948, 1712, 1610, 1546, 1476, 1358, 1295, 1230, 1115, 1067, 808, 742, 680, 538, 462.

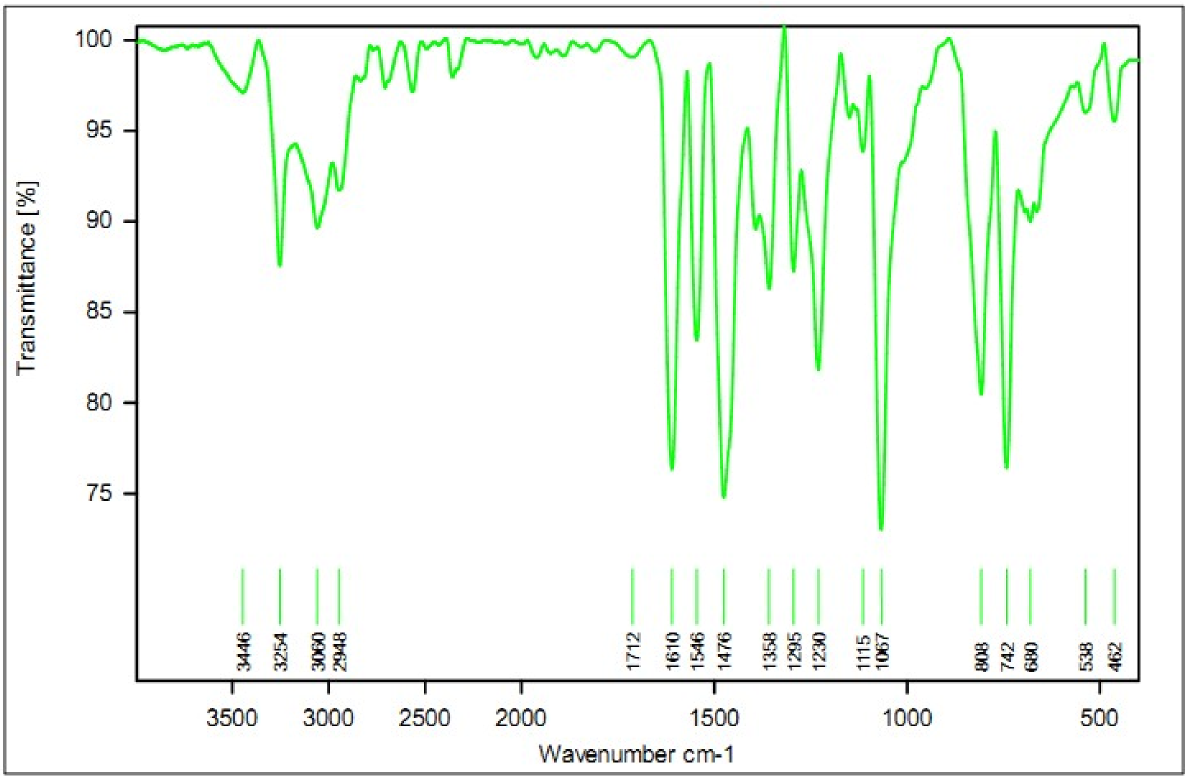

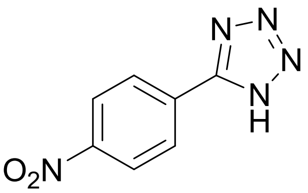

5-(4-nitrophenyl)-1H-tetrazole

^{1}H NMR (300 MHz, DMSO-d6): δ_H= 8.45-8.42 (d, *J*= 6.6 Hz, 2H), 8.31-8.28 (d, *J*= 8.7 Hz, 2H) ppm.

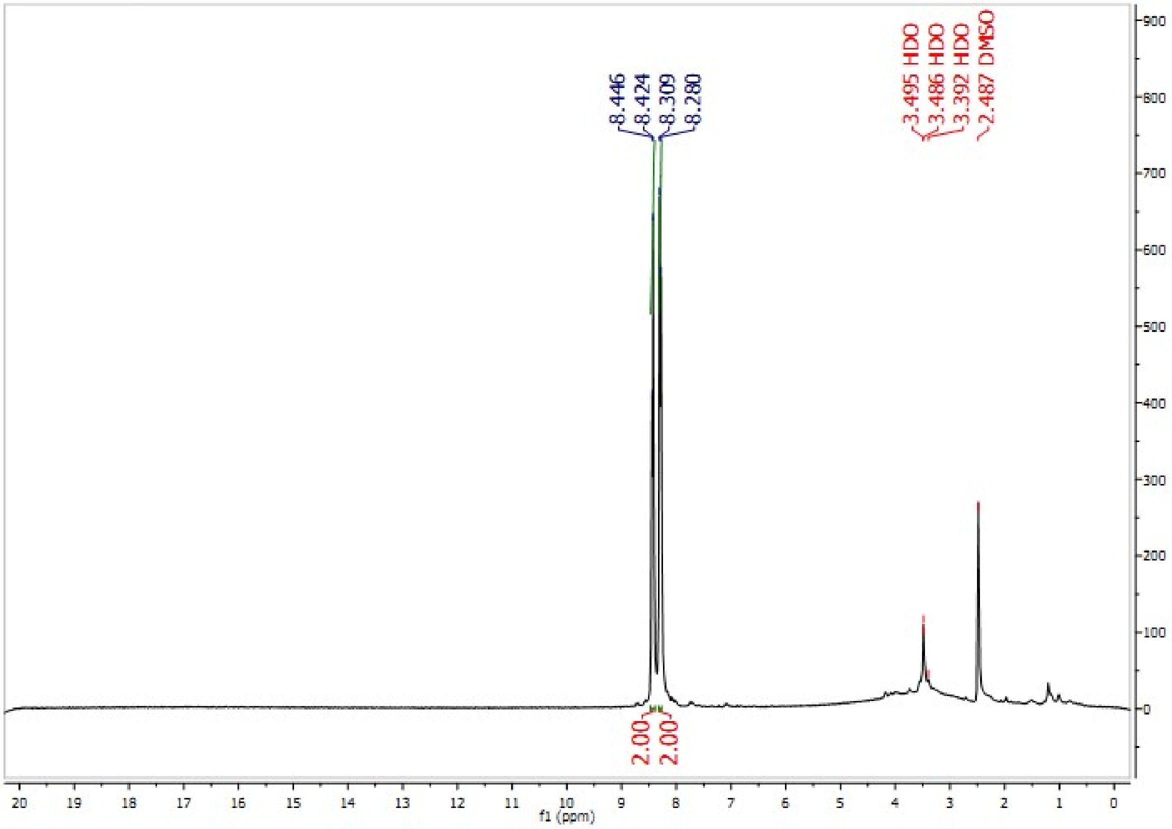

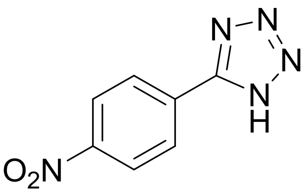

5-(4-nitrophenyl)-1H-tetrazole

^{13}C NMR (100 MHz, DMSO-d6): δ_C= 149.1, 141.2, 131.1, 128.6, 125.0 ppm.

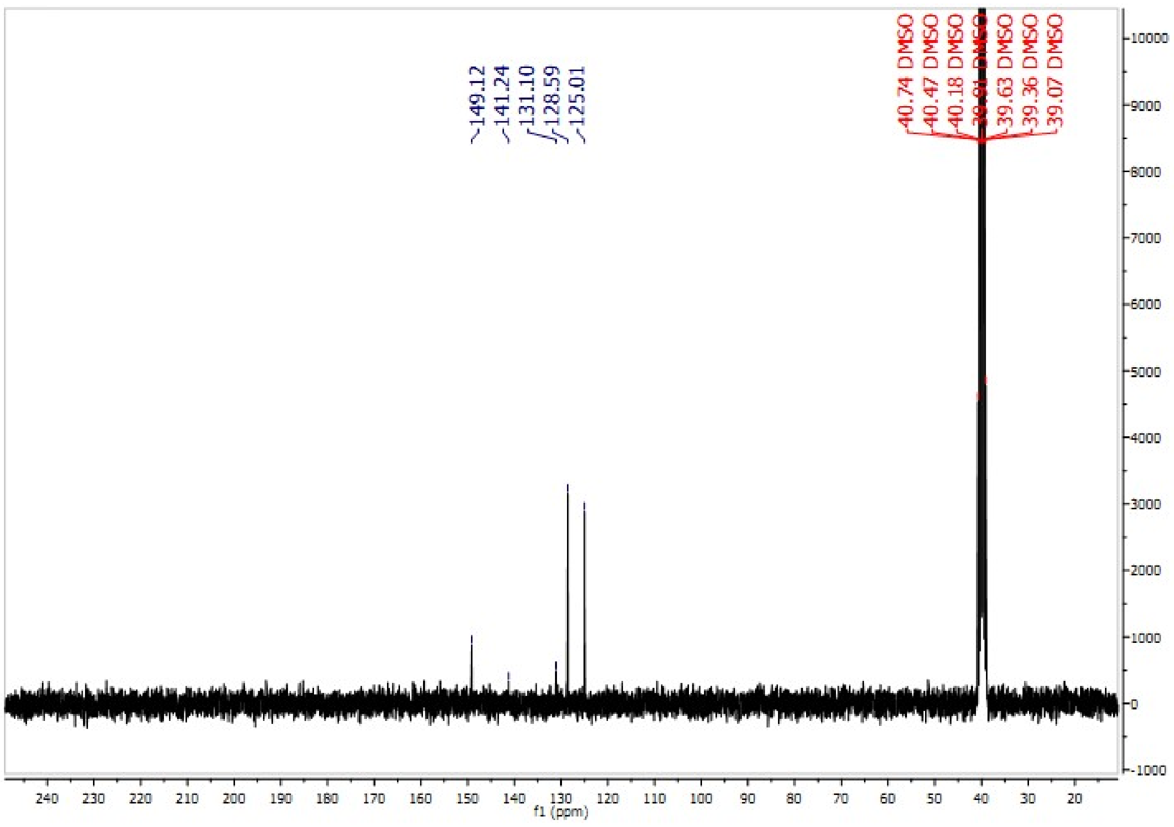

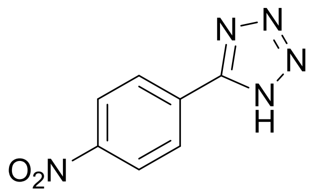

5-(4-nitrophenyl)-1H-tetrazole

IR (KBr) cm^{-1}: 3096, 2850, 1710, 1605, 1550, 1515, 1338, 1286, 1110, 1065, 996, 862, 778, 732, 694, 532, 494, 441.

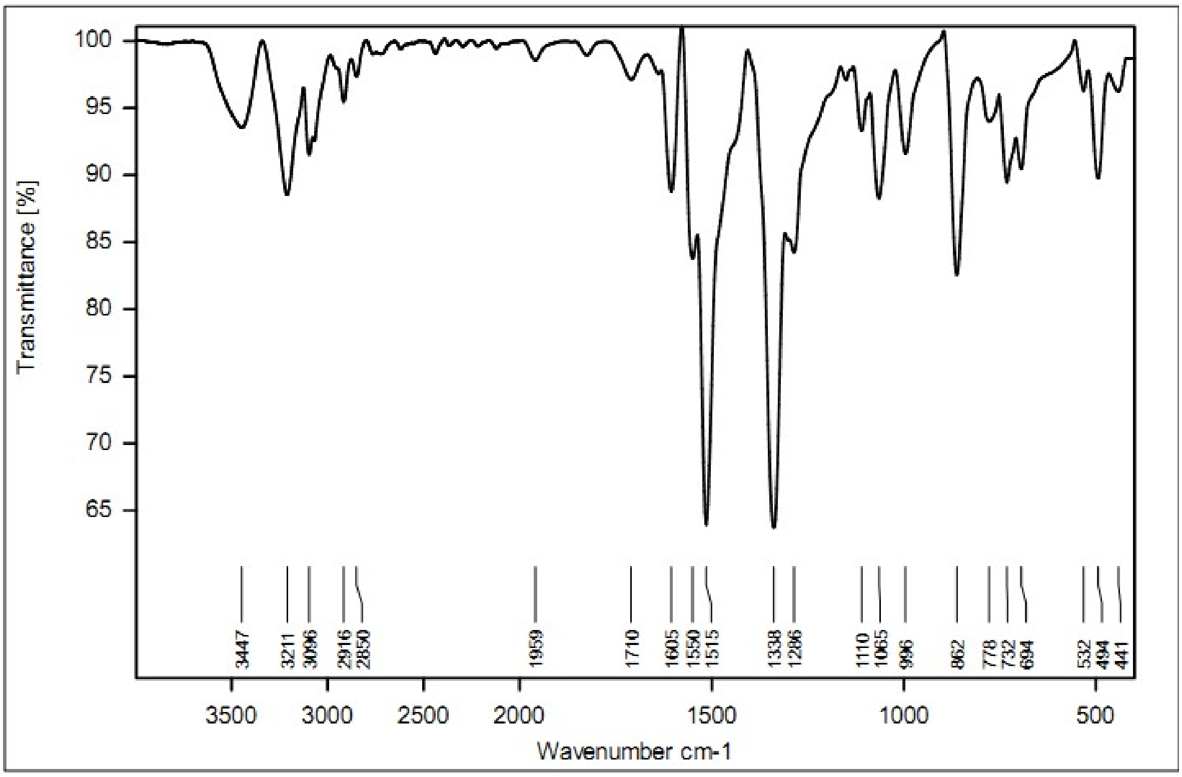

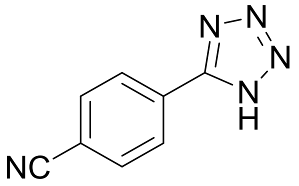

4-(1H-tetrazol-5-yl)benzonitrile

^{1}H NMR (300 MHz, DMSO-d6): δ_H= 8.22-8.19 (d, *J*= 8.4 Hz, 2H), 8.10-8.07 (d, *J*= 8.7 Hz, 2H) ppm.

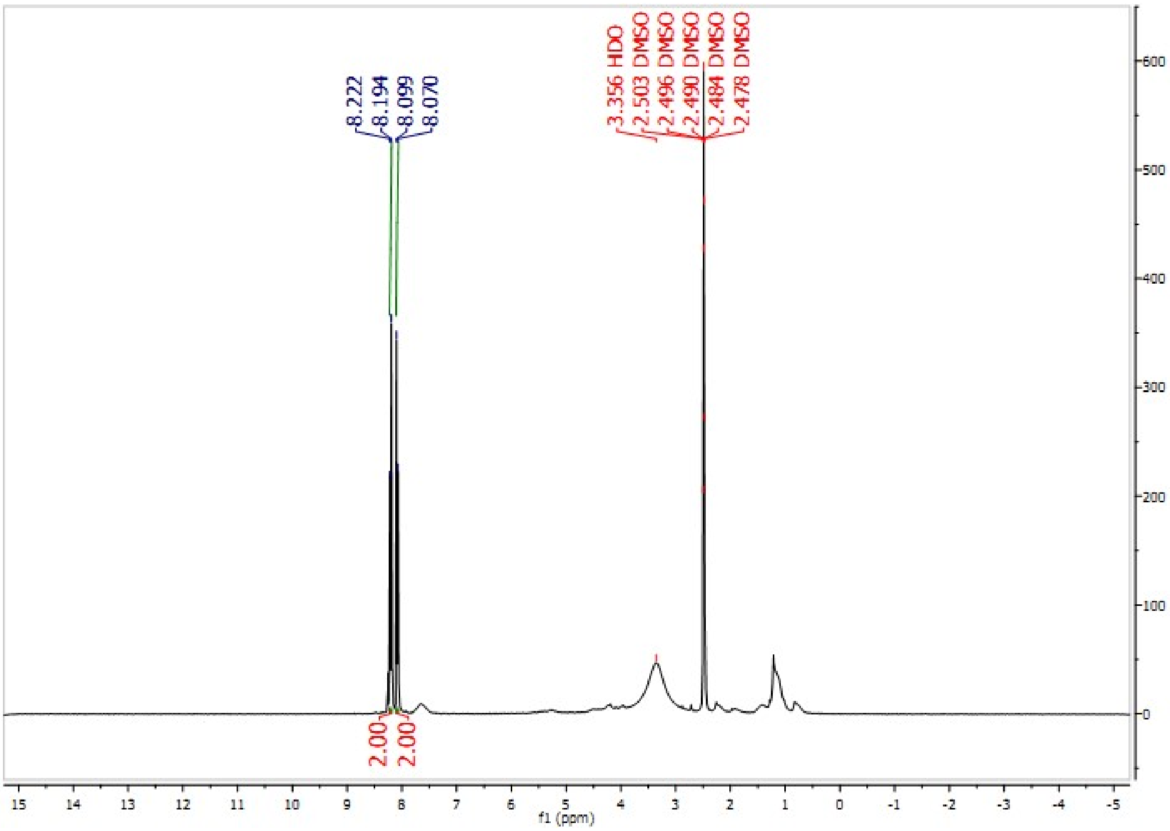

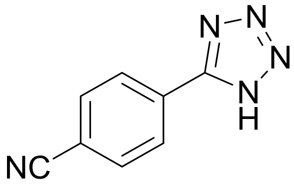

4-(1H-tetrazol-5-yl)benzonitrile

^{13}C NMR (100 MHz, DMSO-d6): δ_C= 138.3, 133.7, 129.4, 128.0, 118.6, 113.7 ppm.

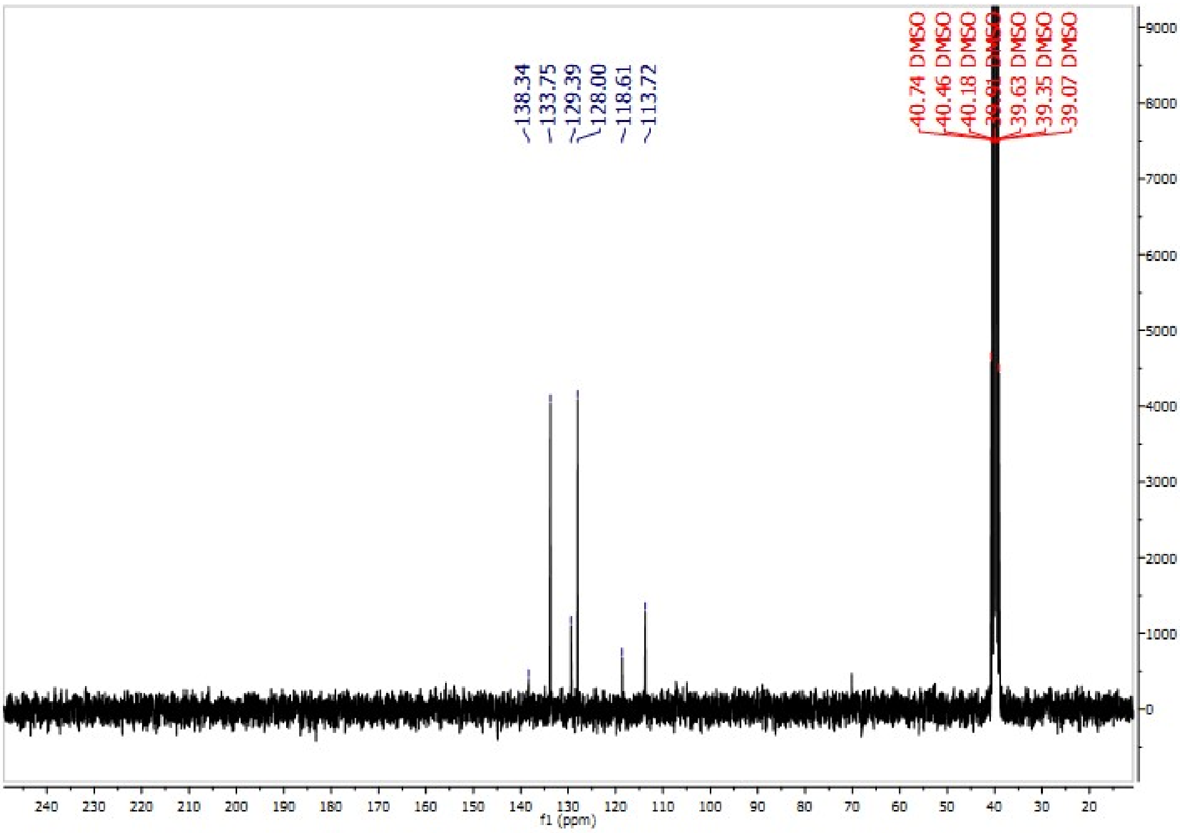

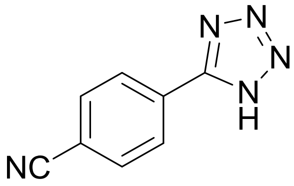

4-(1H-tetrazol-5-yl)benzonitrile

IR (KBr) cm^{-1}: 3476, 3092, 3011, 2923, 2861, 2730, 2623, 2231, 1939, 1727, 1622, 1568, 1497, 1432, 1363, 1277, 1205, 1088, 1029, 987, 845, 751, 701, 605, 555.

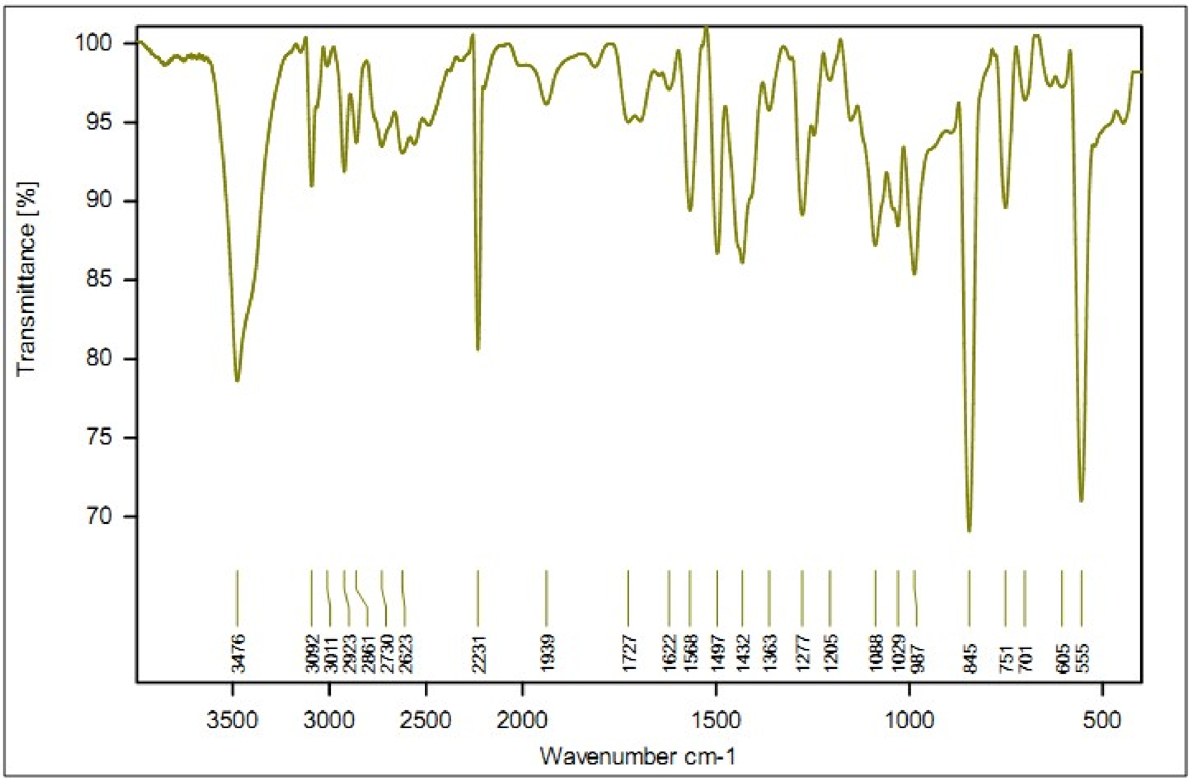

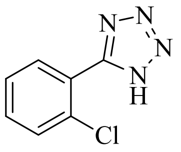

5-(2-chlorophenyl)-1H-tetrazole

^{1}H NMR (300 MHz, DMSO-d6): δ_H= 16.96 (br, 1H), 7.81-7.77 (m, 1H), 7.72-7.67 (m, 1H), 7.65-7.51 (m, 2H) ppm.

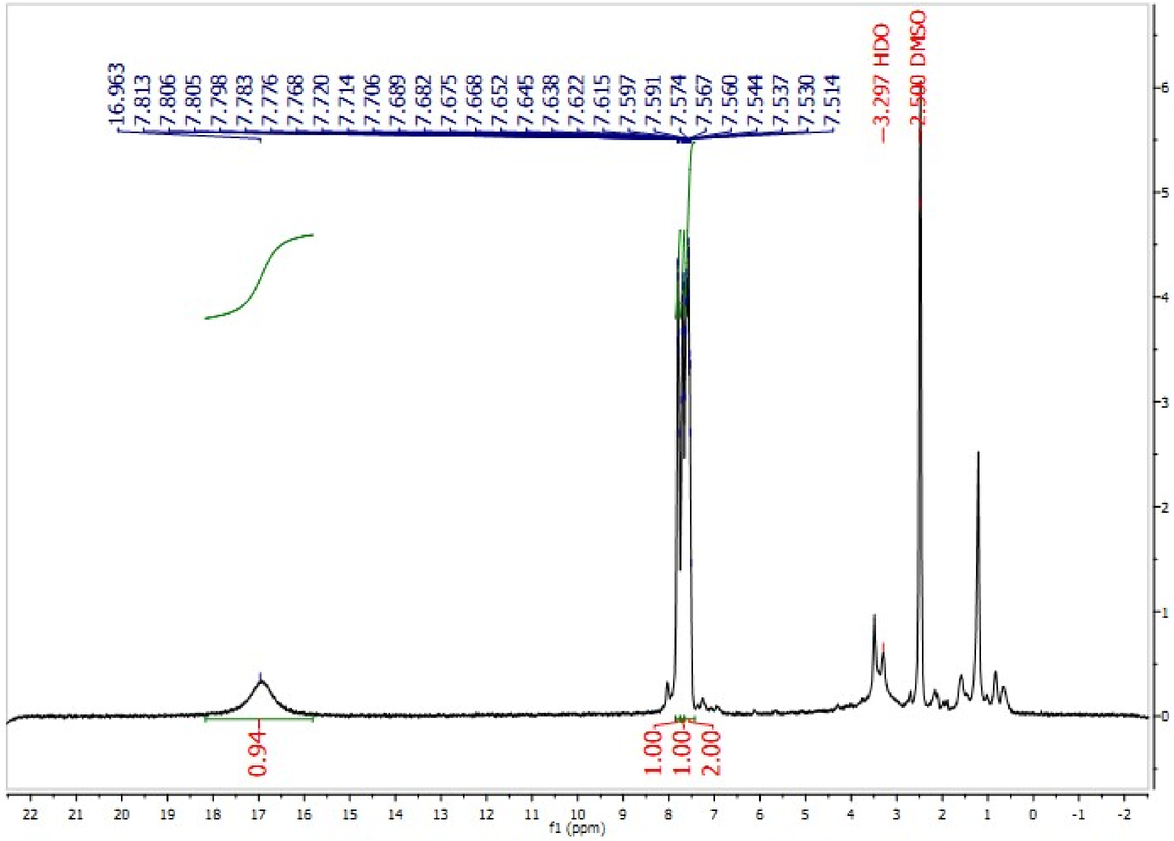

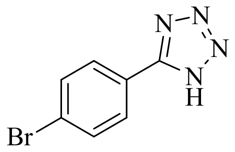

5-(4-bromophenyl)-1H-tetrazole

^{1}H NMR (300 MHz, DMSO-d6): δ_H= 16.91 (br, 1H), 7.97-7.95 (d, *J*= 8.4 Hz, 2H), 7.83-7.80 (d, *J*= 8.4 Hz, 2H) ppm.

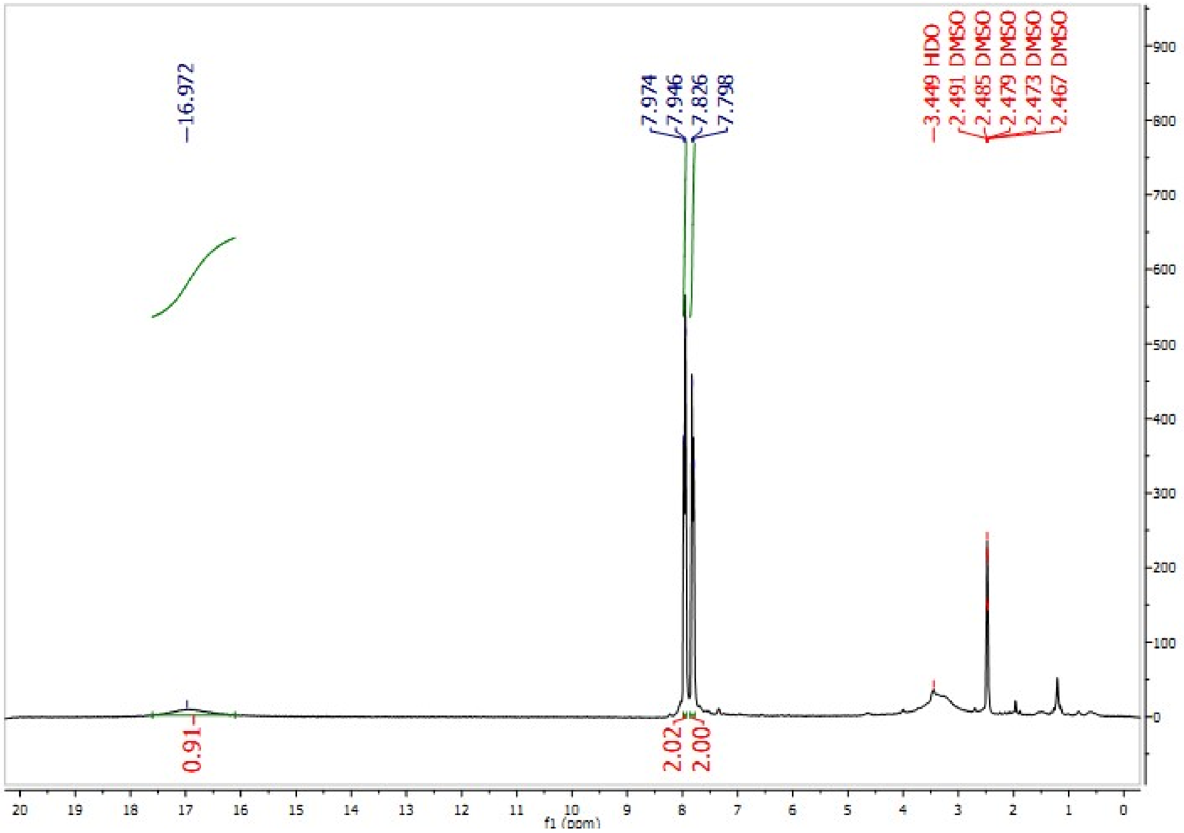

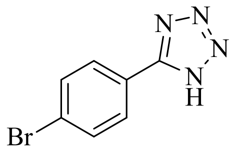

5-(4-bromophenyl)-1H-tetrazole

^{13}C NMR (100 MHz, DMSO-d6): δ_C= 163.5, 128.3, 124.7, 120.5, 119.4 ppm.

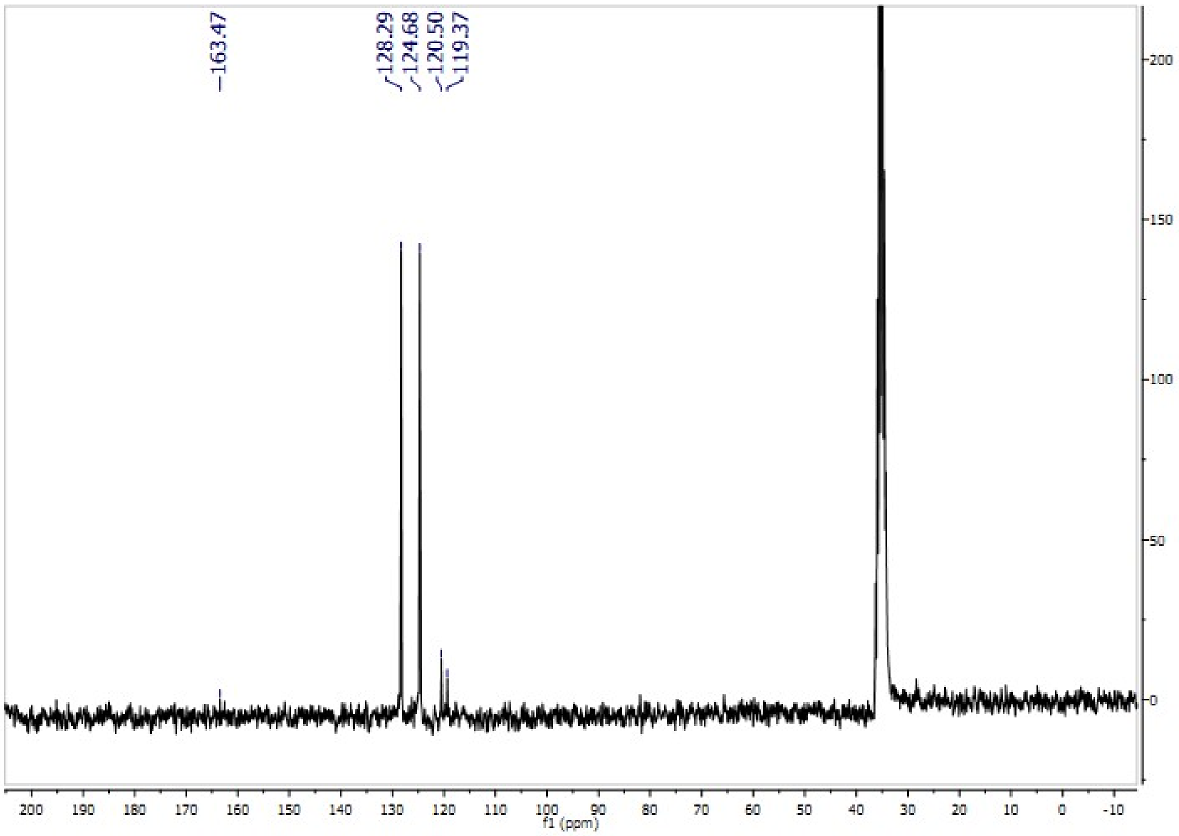

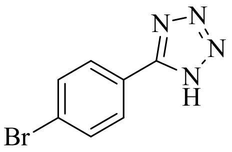

5-(4-bromophenyl)-1H-tetrazole

IR (KBr) cm^{-1}: 3855, 3435, 3081, 2919, 2855, 2726, 1909, 1721, 1604, 1478, 1429, 1269, 1154, 1061, 991, 873, 830, 740, 598, 500, 453.

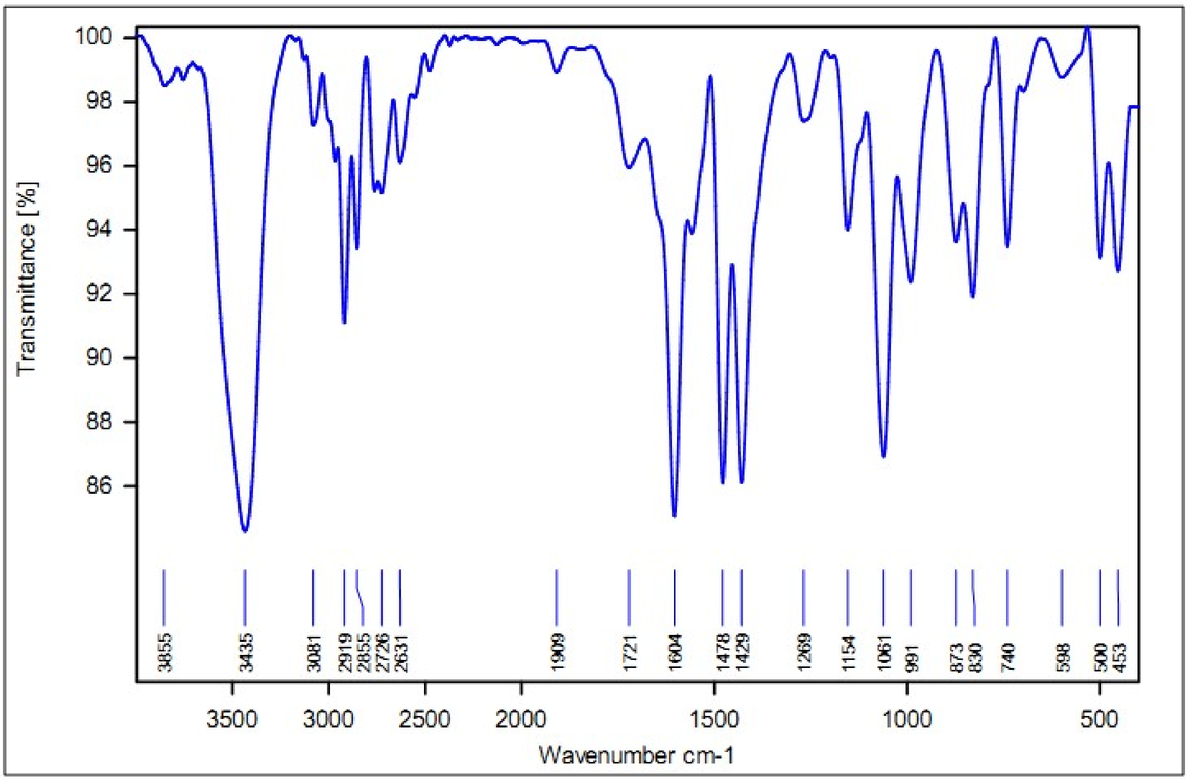

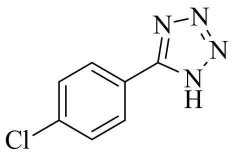

5-(4-chlorophenyl)-1H-tetrazole

^{1}H NMR (300 MHz, DMSO-d6): δ_H= 8.05-8.02 (d, *J*= 9.9 Hz, 2H), 7.69-7.66 (d, *J*= 9.9 Hz, 2H) ppm.

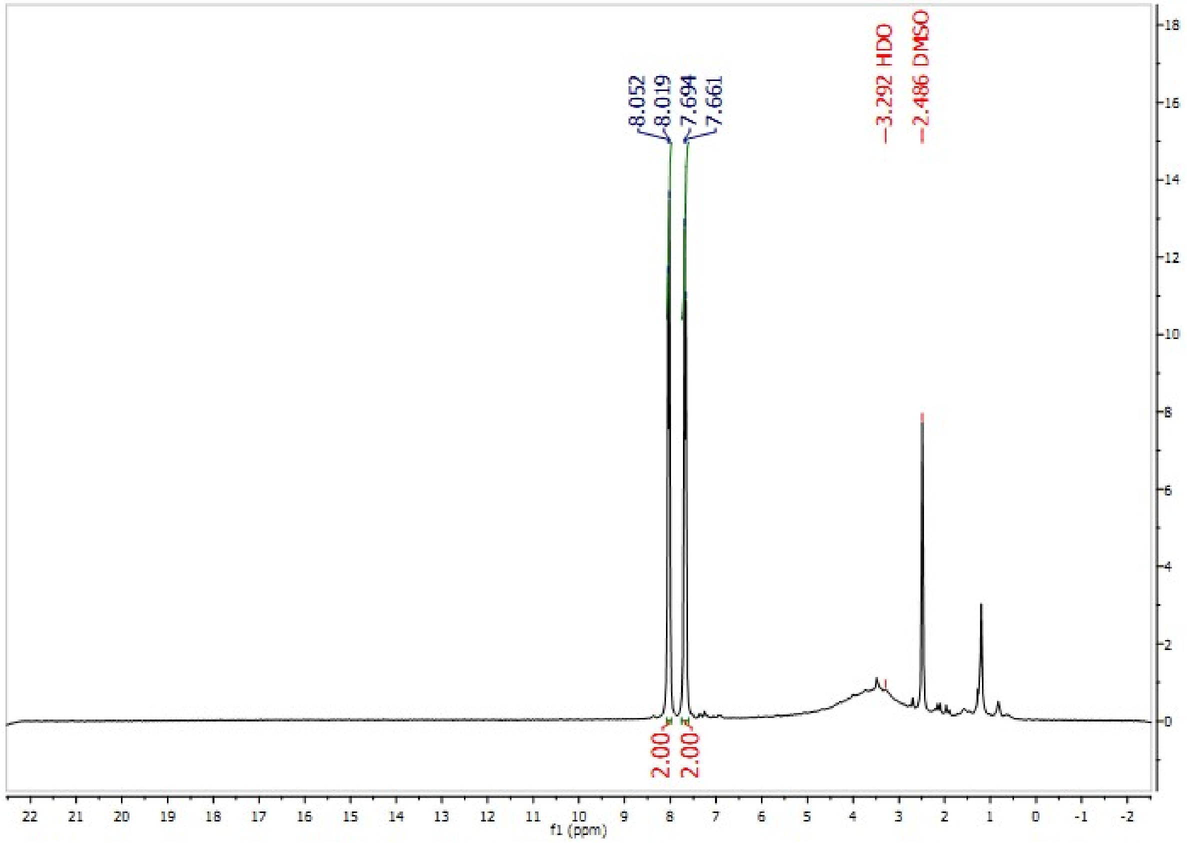

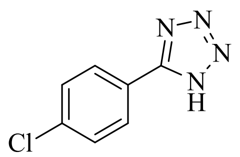

5-(4-chlorophenyl)-1H-tetrazole

^{13}C NMR (100 MHz, DMSO-d6): δ_C= 150.7, 131.7, 125.4, 124.5, 119.0 ppm.

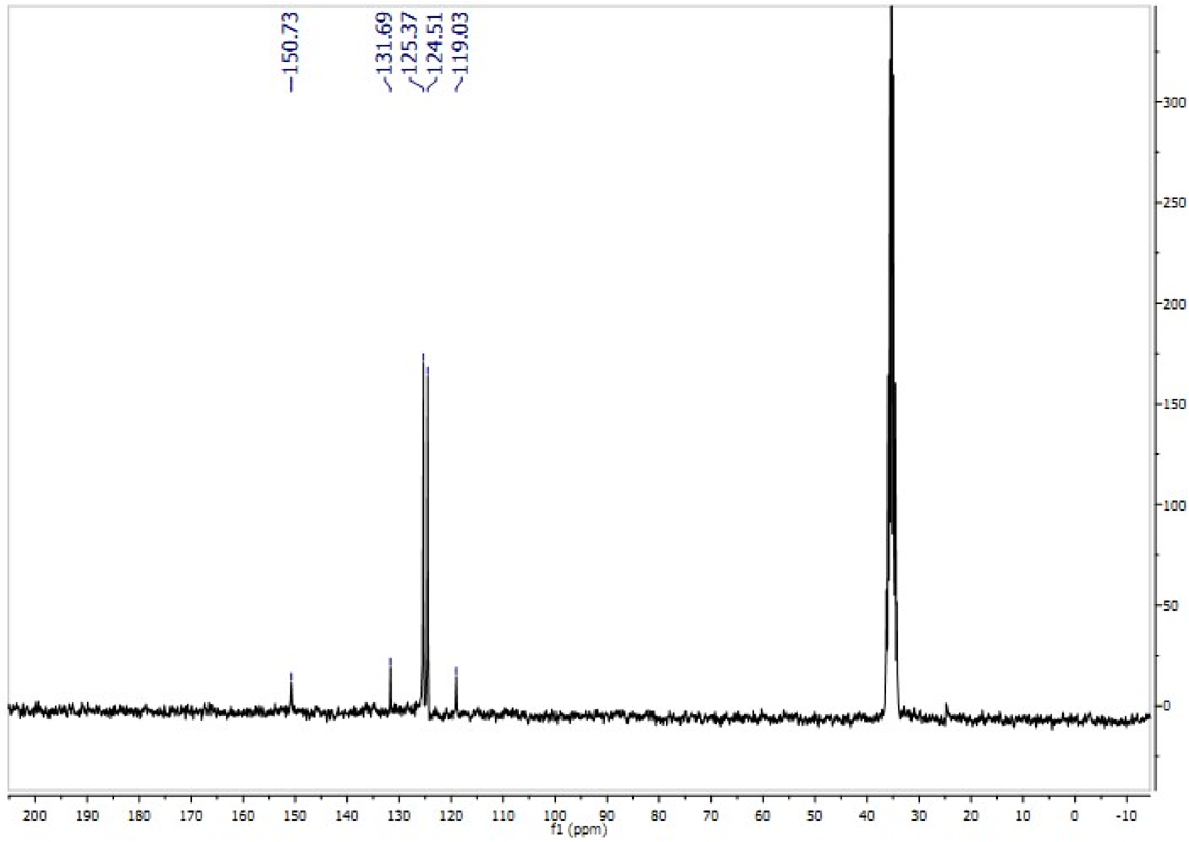

Supplement: NA-006-D3NA01087B-s001 [file NA-006-D3NA01087B-s001.pdf]
